# Barriers and facilitators to care for agitation and/or aggression among persons living with dementia in long-term care

**DOI:** 10.1186/s12877-024-04919-0

**Published:** 2024-04-11

**Authors:** Britney Wong, Zahinoor Ismail, Jennifer Watt, Jayna Holroyd-Leduc, Zahra Goodarzi

**Affiliations:** 1 Department of Community Health Sciences, University of Calgary, Calgary, AB Canada; 2 Cumming School of Medicine, University of Calgary, Calgary, AB Canada; 3 Hotchkiss Brain Institute, University of Calgary, Calgary, AB Canada; 4 Department of Clinical Neurosciences, University of Calgary, Calgary, AB Canada; 5 Department of Psychiatry, University of Calgary, Calgary, AB Canada; 6 Division of Geriatric Medicine, University of Toronto, Toronto, ON Canada; 7 Department of Medicine, University of Toronto, Toronto, ON Canada; 8 Department of Medicine, University of Calgary and Alberta Health Services, Calgary, AB Canada; 9 O’Brien Institute of Public Health, University of Calgary, Calgary, AB Canada

**Keywords:** Dementia, Long-term care, Qualitative, Agitation, Aggression

## Abstract

**Background:**

Agitation and/or aggression affect up to 60% of persons living with dementia in long-term care (LTC). It can be treated via non-pharmacological and pharmacological interventions, but the former are underused in clinical practice. In the literature, there is currently a lack of understanding of the challenges to caring for agitation and/or aggression among persons living with dementia in LTC. This study assesses what barriers and facilitators across the spectrum of care exist for agitation and/or aggression among people with dementia in LTC across stakeholder groups.

**Methods:**

This was a qualitative study that used semi-structured interviews among persons involved in the care and/or planning of care for people with dementia in LTC. Participants were recruited via purposive and snowball sampling, with the assistance of four owner-operator models. Interviews were guided by the Theoretical Domains Framework and transcribed and analyzed using Framework Analysis.

**Results:**

Eighteen interviews were conducted across 5 stakeholder groups. Key identified barriers were a lack of agitation and/or aggression diagnostic measures, limited training for managing agitation and/or aggression in LTC, an overuse of physical and chemical restraints, and an underuse of non-pharmacological interventions. Facilitators included using an interdisciplinary team to deliver care and having competent and trained healthcare providers to administer non-pharmacological interventions.

**Conclusions:**

This study advances care for persons living with dementia in LTC by drawing attention to unique and systemic barriers present across local and national Canadian LTC facilities. Findings will support future implementation research endeavours to eliminate these identified barriers across the spectrum of care, thus improving care outcomes among people with dementia in LTC.

**Supplementary Information:**

The online version contains supplementary material available at 10.1186/s12877-024-04919-0.

## Background

Up to 60% of persons living with dementia (PLWD) in LTC experience agitation and/or aggression symptoms, with the prevalence varying based on dementia pathology and severity [1]. Although agitation and aggression are separate constructs, they are often presented together among PLWD in LTC [2]. Agitation consist of three main domains outlined by the International Psychogeriatric Association criteria for Agitation in Cognitive Disorders: 1) excessive motor activity, 2) verbal aggression, and 3) physical aggression [3]. Aggression refers to verbal and physical behaviours (e.g. hitting, throwing, etc.) that are highly likely to cause harm among the resident and others [4–6]. Agitation and/or aggression can adversely impact residents’ quality of life by increasing the likelihood of falls, fractures, and developing additional neuropsychiatric symptoms, as well as increasing the cost of care and the rate of institutionalization [1, 7]. Corresponding caregivers often experience increased burnout, lower job satisfaction, stress, and worse psychological health [8]. Given the burden that agitation and/or aggression have among PLWD in LTC and their caregivers, more emphasis is needed on treatment strategies.

Agitation and/or aggression can be treated via either pharmacological (drug) or non-pharmacological (non-drug) interventions. The former consists of psychotropic medications, such as antipsychotics (e.g. risperidone or olanzapine) or antidepressants (e.g. citalopram). However, psychotropic medications can lead to adverse side effects including stroke and decreased cognitive function [9, 10]. For example, antipsychotics confer a higher risk of adverse events, such as falls, fractures and deaths [11]. Moreover, the efficacy of psychotropic medications to alleviate agitation symptoms is contested [9]. In comparison, non-pharmacological approaches include sensory practices (e.g., aromatherapy), psychosocial practices (e.g., validation therapy), and structured care protocols (e.g., bathing) [12]. Non-pharmacological approaches are considered first-line treatment strategies to address agitation among PLWD because they confer less side effects and are efficacious [13]. For example, Watt et al. (2019) ranked outdoor activities as highest in efficacy to address combined aggression and agitation, along with physical aggression independently [11]. Despite this knowledge, non-pharmacological treatment approaches are under-used in clinical practice [9].

Many challenges exist to managing agitation and/or aggression in LTC, but prior qualitative studies focus on understanding only nursing and physician perspectives, and lack qualitative perspective on the care needs of PLWD experiencing agitation and/or aggression [9, 14]. As such, there is a lack of understanding on the perceived barriers and facilitators to care for other key stakeholders involved in the care and/or planning of care for PLWD in LTC (e.g., patients, families, allied healthcare workers, etc.). The purpose of this study is to assess what barriers and facilitators to care exist for agitation and/or aggression among PLWD in LTC centres, as perceived by all key stakeholder groups.

## Methods

### Ethics

Ethics approval was acquired through the Conjoint Health Research Ethics Board (REB-22–1100), and permission was granted from all organizations.

### Participants

The sampling frame consisted of persons involved in the care or planning of care of PLWD in LTC. Specifically: (1) physicians (e.g., family doctors, psychiatrists, and geriatricians), (2) nurse practitioners, (3) administrators and decision makers, (4) nursing staff (e.g., registered nurses, licensed practical nurses), (5) allied healthcare workers (e.g., recreational therapists, occupational therapists, physiotherapists, social workers), (6) care aides, and (7) LTC residents and family members. Participants were all 18 years or older, and English-speaking. No other restrictions were used.

### Participant recruitment

Rolling, snowball and purposive sampling of participants were used, with the latter ensuring representation of diverse sex, gender, race/ethnicity, and urban/rural perspectives. We recruited persons working across healthcare disciplines (e.g., physicians, nurses, allied healthcare workers, etc.). 70% of the total resident population, and an estimated 89% of care staff, are female in LTC [15, 16]. Therefore, to ensure fair representation, male participants were purposively recruited across all disciplines [15]. Participants were recruited from urban, suburban and rural sites to increase understanding of the organizational differences and similarities between geographical regions.

Recruitment posters and email advertisements were sent out across four local LTC facilities. The study team further recruited participants via our own networks. To ensure representation at a national level, advertisements were posted to social media platforms (e.g., Twitter). Lastly, persons who consented to participate in a previous Delphi panel study developing a novel care pathway for agitation and/or aggression among PLWD in LTC could also consent to being contacted about participating in a semi-structured interview [17]. In the Delphi study, panelists were recruited to complete several rounds of a Delphi survey to create a clinical care pathway suitable for the identification, diagnosis, and management of agitation and/or aggression symptoms among PLWD in LTC [17]. Panelists were recruited via the same 4 LTC centres via purposive and snowball sampling using poster and email advertisements [17]. They were also recruited via research team contacts and networks [17].

Semi-structured interviews (45 to 60 min) were conducted one-on-one with participants until thematic saturation was reached. No compensations were offered to participants of the study.

### Data collection, storage and management

Interviews occurred online using a password-protected meeting using the platform “Zoom” in a confidential environment. Participants’ personal information was not shared outside of the research team. All interviews were conducted, transcribed, and verified by one researcher. The interviewer is a cis female graduate student. The interviews were audio recorded using an audio recorder, de-identified using pseudonyms, and transcribed verbatim using the A.I software “Otter.ai”. If audio recordings had identifying information, they were transcribed by hand. Each transcript was verified against the corresponding audio recordings for accuracy. All original recording files will be kept on the password-protected university server for a minimum of 5 years following transcription, in accordance with [redacted] data retention policies.

### Interview guide development

Interview guides were developed by the research team based on existing evidence, expert experience and framed with the Theoretical Domains Framework (TDF), as it identifies influences on healthcare providers’ and patients’ behaviours relative to evidence-based recommendations [18]. The TDF was chosen over other frameworks because it comprehensively captures a range of mechanisms that influence behaviours, creating a foundation for prospective behaviour change interventions [19]. The TDF can be mapped to the Capacity, Opportunity, Motivation Model of Behaviour (COM-B) within the Behaviour Change Wheel (BCW) [20]. The COM-B can then be used as a stepping stone to link these sources of behaviour to behaviour change interventions and clinical implementation [21].

Two separate interview guides were created for: 1) healthcare practitioners (e.g., physicians, nurses, allied healthcare workers) and; 2) caregivers and family members. Questions covered all 14 domains of the TDF (e.g., knowledge, skills, etc.). The interview guides can be found in Additional File 2. The aforementioned definitions of agitation and aggression were followed when creating the interview guide. Barriers/facilitators that may exist at diagnosis/detection, care management and coordination, and treatment (mild/moderate and severe/acute) of agitation and/or aggression were explored among PLWD in LTC. The guide was adapted for suitability and/or appropriateness to ensure both caregivers and healthcare practitioners could answer.

### Data analysis

#### Descriptive statistics

Demographic data was summarized from all interview participants. Characteristics included sex, gender, age, place of birth, languages spoken, racial identity, occupation or role in LTC, and length of career or number of years in their role. These data were reported in a table, providing rich, descriptive context of the interview participants overall (Table 1).

**Table 1:** The Demographic of Semi-Structured Interview Participants (*n* = 18)

Demographic Question		Number of Participants n(%)
**Sex**	Female	15(83.3%)
	Male	3(16.67%)
**Gender**	Woman	15(83.3%)
	Man	3(16.67%)
**Age Group**	18–34	3(16.67%)
	35–49	6(33.3%)
	50–64	6(33.3%)
	65–84	3(16.67%)
	85 +	0(0.0%)
**Birth Place**	Canada	15(83.3%)
	Philippines, Zimbabwe, Germany	3(16.67%)
**Languages Spoken**	English	18(100.0%)
	French	2(11.1%)
	Tagalog, Cantonese, Shona	3(16.67%)
**Racial Identity**	African/Black, Middle Eastern	2(11.1%)
	Caucasian/White	15(83.3%)
	Southeast Asian, Chinese	2(11.1%)
**Roles in LTC**	Family caregivers, spouses	5(27.8%)
	Family physicians	4(22.2%)
	Nurses (RNs, LPNs) and Healthcare Aides	5(27.8%)
	Executive Medical Directors, Quality Practice Leads	2(11.1%)
	Other Allied Healthcare Workers (OT, RT, Spiritual Care Practitioner)	5(27.8%)
**Number of years in role**	0–5	5(27.8%)
	6–10	5(27.8%)
	11–15	0(0.0%)
	16 +	8(44.4%)

#### Framework analysis

The transcribed interviews were coded using Framework Analysis, based on the TDF. Framework analysis determined how interview discussions fit within the TDF. It follows 7 steps described by Gale et al [22].

An inductive, ground-up coding process was conducted by two independent researchers by analyzing each line of transcript one-by-one. Codes emerged as the data were analyzed. Codes were then deductively analyzed by one researcher, by grouping them into themes and assigning TDF domains to them. Each code could be associated with one or more TDF domain. The themes were further grouped into categories of care for agitation and/or aggression: 1) Detection/diagnosis, 2) Care coordination and management, and; 3) Treatment (mild/moderate and acute/severe). Further interpretation was made on what domains of the TDF were contributing the most as barriers/facilitators to care.

Data saturation was considered reached when no new themes regarding barriers and facilitators to agitation care emerged from the discussions [18]. As new themes continued to arise with coding, more participants from the respective stakeholders were recruited via purposive and snowball sampling until data saturation was achieved, and possible themes were exhausted.

### Reporting criteria

Results were reported as per the 32-item COREQ checklist for explicit reporting of qualitative studies involving semi-structured interviews [23]. A reflexivity statement is shown in Additional File 1.

## Results

### Participant information

Semi-structured interviews were conducted between December 2022 and February 2023. 18 participants were interviewed across the 4 LTC centres, with the majority being female (*n* = 15), between the ages of 35–64, born in Canada (*n* = 15), White (*n* = 15) and English-speaking (*n* = 18) (Table 1). Participants held a variety of roles within LTC: family caregivers and spouses (*n* = 5), family physicians (*n* = 4), nurses (registered nurses, licensed practical nurses) (*n* = 4), healthcare aides, executive medical directors and quality practice leads (*n* = 4), and other allied healthcare workers (i.e., recreational therapists, occupational therapists and spiritual care practitioners) (*n* = 5).

### Organization of Findings

**Fig. 1:**
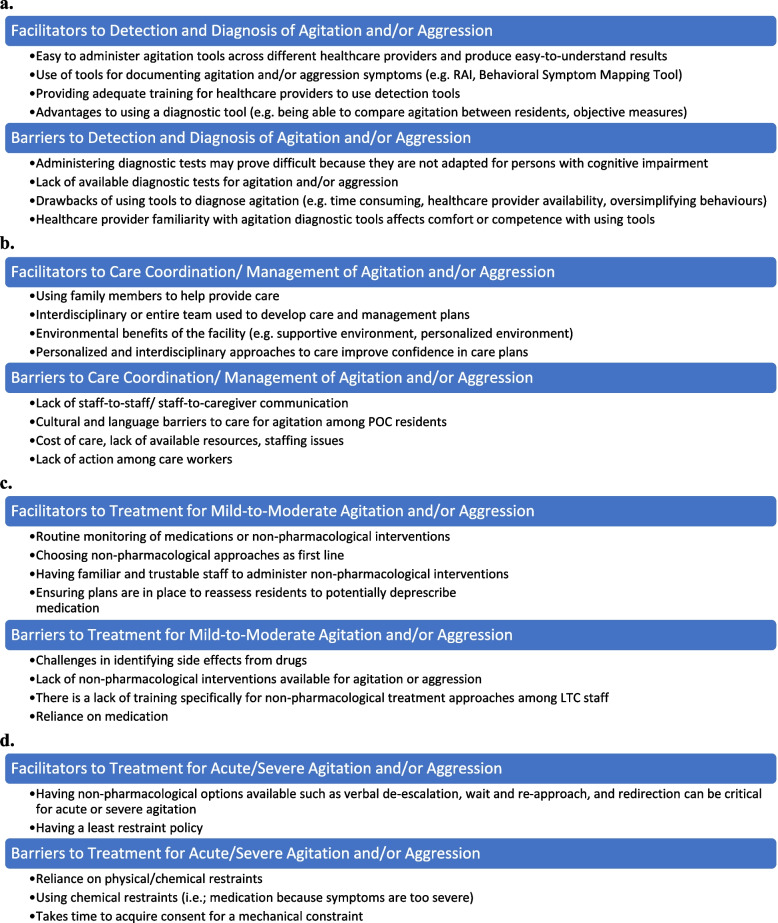
The most common facilitators and barriers to (**a**) the detection and diagnosis of agitation and/or aggression in LTC, (**b**) the care coordination and management of agitation and/or aggression in LTC, (**c**) the treatment of mild-to-moderate agitation and/or aggression in LTC, and (**d**) the treatment of acute/severe agitation and/or aggression in LTC

Results are presented as barriers and/or facilitators across several larger categories (Fig. 1): (1) detection and diagnosis, (2) Care coordination and management, (3) Mild-to-moderate Treatment, and (4) Acute/Severe treatment. Themed codes and associated interviewee quotes are indicated by italics as shown below. Tables 2, 3 and 4 demonstrate all codes and categorized themes which depict all barriers and facilitators to care identified during the interviews, with detailed quotes in Additional File 3. Participant roles were anonymized to protect participant confidentiality, but Participant ID is shown to represent diverse participant perspectives.

**Table 2:** Codes related to detection and diagnosis of agitation and/or aggression, mapped to the TDF and linked to the COM-B model [24]

COM-B		TDF Domain	Codes	Barrier or Facilitator
Capability	Psychological	Knowledge	Healthcare provider familiarity with agitation diagnostic tools affects comfort or competence with using tools	Barrier
			Difficulties among healthcare providers in understanding how agitation diagnostic tests works	Barrier
			Lack of available diagnostic tests for agitation and/or aggression	Barrier
			Residents are unique and agitation is especially unpredictable and fluctuates over time	Barrier
			Diagnostic practices overlook hypoactive behaviours	Barrier
			No formal criterion for agitation are used	Barrier
			Use of Diagnostic test to diagnose agitation (E.g. RAI)	Facilitator
			Providing adequate training for healthcare providers to use agitation screening tools	Facilitator
			When to involve geriatric medicine or geriatric psychiatry (e.g. on a referral basis)	Facilitator
			Diagnosis for agitation and/or aggression is made during the process of the dementia diagnosis	Facilitator
			Documenting and recording agitation events in many places	Facilitator
			Reviewing experiences of all care team members working with the resident to create a diagnosis of agitation and/or aggression	Facilitator
		Cognitive and Interpersonal skills	Healthcare provider familiarity with agitation diagnostic tools affects comfort or competence with using tools	Barrier
			Difficulties among healthcare providers in understanding how agitation diagnostic tests works	Barrier
			Drawbacks of using tools to diagnose agitation (e.g. time consuming, healthcare provider availability, oversimplifying behaviours)	Barrier
			Residents are unique and agitation is especially unpredictable and fluctuates over time	Barrier
			No formal criterion for agitation are used	Barrier
			Diagnostic practices overlook hypoactive behaviours	Barrier
			Providing adequate training for healthcare providers to use agitation screening tools	Facilitator
			Counting number of aggressive or agitated incidents to diagnose agitation	Facilitator
			When to involve geriatric medicine or geriatric psychiatry (e.g. on a referral basis)	Facilitator
			Documenting and recording agitation events in many places	Facilitator
			Diagnosis for agitation and/or aggression is made during the process of the dementia diagnosis	Facilitator
		Memory, Attention and Decision Making Processes	Administering diagnostic tests may prove difficult because they are not adapted for persons with cognitive impairment	Barrier
		Behavioural Regulation	Diagnostic practices overlook hypoactive behaviours	Barrier
			Residents are unique and agitation is especially unpredictable and fluctuates over time	Barrier
	Physical	Physical Skills	-	-
Opportunity	Social	Social influences	Unclear awareness or availability of geriatric medicine or geriatric psychiatry services	Barrier
	Physical	Environmental Context and Resources	Drawbacks of using tools to diagnose agitation (e.g. time consuming, healthcare provider availability, oversimplifying behaviours)	Barrier
			Diagnosis of cognitive issues takes a long time, which delays diagnosis of agitation and/or aggression	Barrier
			Lack of available diagnostic tests for agitation and/or aggression	Barrier
			Unclear awareness or availability of geriatric medicine or geriatric psychiatry services	Barrier
Motivation	Reflective	Social/Professional Role and Identity	Less referrals needed in LTC centres where physicians are more actively involved in care	Facilitator
			Healthcare provider familiarity with agitation diagnostic tools affects comfort or competence with using tools	Barrier
			Drawbacks of using tools to diagnose agitation (e.g. time consuming, healthcare provider availability, oversimplifying behaviours)	Barrier
			Specialized care teams helped with diagnoses	Facilitator
			Providing adequate training for healthcare providers to use agitation screening tools	Facilitator
			When to involve geriatric medicine or geriatric psychiatry (e.g. on a referral basis)	Facilitator
			Less referrals needed in LTC centres where physicians are more actively involved in care	Facilitator
			Diagnosis is made by a physician	Facilitator
			Unclear awareness or availability of geriatric medicine or geriatric psychiatry services	Barrier
			Reviewing experiences of all care team members working with the resident to create a diagnosis of agitation and/or aggression	Facilitator
		Beliefs about capabilities	Easy to administer agitation tools across different healthcare providers and produce easy-to-understand results	Facilitator
			Advantages to using a diagnostic test (e.g. being able to compare agitation between residents, objective measures)	Facilitator
			Drawbacks to tools to monitor agitation symptoms (e.g. not informative enough)	Barrier
			Preference among healthcare providers for screening tools	Facilitator
		Optimism	Easy to administer agitation tools across different healthcare providers and produce easy-to-understand results	Facilitator
		Beliefs about Consequences	Drawbacks to tools to monitor agitation symptoms (e.g. not informative enough)	Barrier
		Intentions	Use of tools for diagnosing agitation and/or aggression symptoms (e.g. DSM-Ts, daily behavioural mapping, RAI assessment etc.)	Facilitator
			Use of Diagnostic test to diagnose agitation (E.g. RAI)	Facilitator
			Counting number of aggressive or agitated incidents to diagnose agitation	Facilitator
		Goals	Use of tools for diagnosing agitation and/or aggression symptoms (e.g. DSM-Ts, daily behavioural mapping, RAI assessment etc.)	Facilitator
			Use of Diagnostic test to diagnose agitation (E.g. RAI)	Facilitator
			Documenting and recording agitation events in many places	Facilitator
			Counting number of aggressive or agitated incidents to diagnose agitation	Facilitator
	Automatic	Reinforcement	Specialized care teams helped with diagnoses	Facilitator
			Drawbacks to tools to monitor agitation symptoms (e.g. not informative enough)	Barrier
			Unclear awareness or availability of geriatric medicine or geriatric psychiatry services	Barrier
			Diagnosis for agitation and/or aggression is made during the process of the dementia diagnosis	Facilitator
			The high volume of assessments for other behavioural issues is part of the assessment for agitation	Facilitator
			Documenting and recording agitation events in many places	Facilitator
			Reviewing experiences of all care team members working with the resident to create a diagnosis of agitation and/or aggression	Facilitator
		Emotion	Diagnostic practices overlook hypoactive behaviours	Barrier
			Residents are unique and agitation is especially unpredictable and fluctuates over time	Barrier
			Residents' lack of awareness or expression	Barrier

**Table 3:** Codes Related to Care Coordination and Management, Mapped to the TDF and the COM-B Model

COM-B		TDF Domain	Codes	Barrier or Facilitator
Capability	Psychological	Knowledge	Constantly changing directives in LTC facilities for agitation and/or aggression	Barrier
			Lack of directives in LTC facilities for agitation or aggression	Barrier
			Lack of Communication (Between staff and between staff/caregivers)	Barrier
			Lack of competency of staff to deliver care	Barrier
			Lack of Coordination of Care among team members in LTC	Barrier
			Lack of Education among friend and/or family caregivers for caring for agitation among people with dementia	Barrier
			Lack of attention to hypoactive behaviours	Barrier
			Lack of training for healthcare providers caring for agitation or aggression among people with dementia	Barrier
			Inconsistent training for health care providers caring for people with dementia with agitation	Barrier
			Changing language around agitated and aggressive behaviours to be more patient-centred	Facilitator
			Healthcare providers need to be able to self-regulate when providing care to aggressive residents	Facilitator
			Adequate training is provided to increase competency and expertise among LTC staff	Facilitator
			Education of friend and family caregivers enables better agitation and/or aggression care among people with dementia in LTC	Facilitator
			Education among healthcare providers for management approaches for agitation and/or aggression enables better care	Facilitator
			Staff from different cultural backgrounds respond differently to agitation	Barrier
			Different healthcare providers perceive planning of care to be specific to their professional roles	Barrier
			Ensuring that the follow-up of agitated symptoms is integrated into care practices	Facilitator
		Cognitive and Interpersonal skills	Lack of competency among staff to deliver care	Barrier
			Lack of Coordination of Care among team members in LTC	Barrier
			Lack of Education among friend and/or family caregivers for caring for agitation among people with dementia	Barrier
			Staff only have personal background knowledge as their training, with no extra education from long-term care	Barrier
			Lack of attention to hypoactive behaviours	Barrier
			Lack of training for healthcare providers caring for agitation or aggression among people with dementia	Barrier
			Inconsistent training for health care providers caring for people with dementia with agitation	Barrier
			Too many staff members handling a patient resulting in agitation	Barrier
			Healthcare providers need to be able to self-regulate when providing care to aggressive residents	Facilitator
			Adequate training is provided to increase competency and expertise among LTC staff	Facilitator
			Education of friend and family caregivers enables better agitation and/or aggression care among people with dementia in LTC	Facilitator
			Education among healthcare providers for management approaches for agitation and/or aggression enables better care	Facilitator
			Confidence in Care Plan	Facilitator
			Checklist of precipitants to consider (e.g. basic needs: food, drink, pain, medication, etc.)	Facilitator
			Different healthcare providers perceive planning of care to be specific to their professional roles	Barrier
			Staff from different cultural backgrounds respond differently to agitation	Barrier
		Memory, Attention and Decision Making Processes	Decline in mental status or increased dementia impeding care for agitation	Barrier
			Resident personal qualities as barrier to care	Barrier
			Residents having difficulty communicating needs	Barrier
			It is important to look for triggers, contributing causes, and unmet needs that lead to agitation and/or aggression	Barrier
			Residents respond better to some staff members and disciplines than others (e.g. rec therapy)	Facilitator
		Behavioural Regulation	Lack of attention to hypoactive behaviours	Barrier
			Difficulties among LTC staff to understand the residents’ needs (e.g. likes, dislikes)	Barrier
			Resident personal qualities as barrier to care	Barrier
			Healthcare providers are not communicating care practices with residents	Barrier
			Appropriate Use of Antipsychotics as helpful for pharmacological use guidelines	Facilitator
			Having a checklist of precipitants to consider (e.g. basic needs: food, drink, pain, medication, etc.) aids in management plans	Facilitator
			Care plans for all interventions need to be tailored and patient-centred	Facilitatorgivi
			Residents respond better to some staff members and disciplines than others (e.g. rec therapy)	Facilitator
	Physical	Physical Skills	-	-
Opportunity	Social	Social influences	Healthcare practitioners are not raising awareness of issues for fear of job (e.g. termination, etc.)	Barrier
			High staff turnover in LTC	Barrier
			There is a lack of personalized care plans and thus low confidence in care	Barrier
			Lack of attention to hypoactive behaviours	Barrier
			Inconsistent training for health care providers caring for people with dementia with agitation	Barrier
			Not enough time for health care providers to provide care	Barrier
			Certain staff members unable to carry out care due to personal characteristics	Barrier
			Hiring someone to carry out interventions or day to day affairs with resident	Facilitator
			Communication with staff is effective among persons involved with the care or planning of care in LTC	Facilitator
			Hiring recreational therapist privately for residents with agitation	Facilitator
			Having a checklist of precipitants to consider (e.g. basic needs: food, drink, pain, medication, etc.) aids in management plans	Facilitator
			Residents respond better to some staff members and disciplines than others (e.g. rec therapy)	Facilitator
			Staff from different cultural backgrounds respond differently to agitation	Barrier
	Physical	Environmental Context and Resources	Constantly changing directives in LTC facilities for agitation and/or aggression	Barrier
			Lack of directives in LTC facilities for agitation or aggression	Barrier
			Cost of care barriers	Barrier
			Cultural Barriers to Care for Agitation among POC residents	Barrier
			Environmental Challenges (e.g. loud noises, unideal room configurations)	Barrier
			Lack of Communication (Between staff and between staff/caregivers)	Barrier
			Lack of communication between health facilities	Barrier
			Lack of Education among friend and/or family caregivers for caring for agitation among people with dementia	Barrier
			Healthcare practitioners are not raising awareness of issues for fear of job (e.g. termination, etc.)	Barrier
			High staff turnover in LTC	Barrier
			There is a lack of personalized care plans and thus low confidence in care	Barrier
			Inconsistent training for health care providers caring for people with dementia with agitation	Barrier
			Not enough time for health care providers to provide care	Barrier
			Reliance on caregiver as management strategy for agitation	Barrier
			Lack of available resources	Barrier
			Too many staff members handling a patient resulting in agitation	Barrier
			We can identify an unmet need, but there can be difficulty with solving it	Barrier
			Hiring someone to carry out interventions or day to day affairs with resident	Facilitator
			Environmental Benefits of the facility (e.g. supportive environment, personalized environment)	Facilitator
			Hiring recreational therapist privately for residents with agitation	Facilitator
			Having a checklist of precipitants to consider (e.g. basic needs: food, drink, pain, medication, etc.) aids in management plans	Facilitator
			Lack of Communication between LTC centres	Barrier
Motivation	Reflective	Social/Professional Role and Identity	Lack of action among care workers	Barrier
			Caregivers may not always understand how agitation and/or aggression impacts patient care	Barrier
			Caregivers may under-report symptoms	Barrier
			Lack of Communication (Between staff and between staff/caregivers)	Barrier
			Lack of communication between health facilities	Barrier
			Lack of competency of staff to deliver care	Barrier
			Lack of Coordination of Care among team members in LTC	Barrier
			Too many staff members handling a patient resulting in agitation	Barrier
			Lack of Education among friend and/or family caregivers for caring for agitation among people with dementia	Barrier
			Staff only have personal background knowledge as their training, with no extra education from long-term care	Barrier
			Healthcare practitioners are not raising awareness of issues for fear of job (e.g. termination, etc.)	Barrier
			Lack of Inclusion of Needs and Values of family and residents	Barrier
			Reliance on caregiver as management strategy for agitation	Barrier
			Certain staff members unable to carry out care due to personal characteristics	Barrier
			Staffing issues	Barrier
			Using family members to help provide care	Facilitator
			Communication with staff is effective among persons involved with the care or planning of care in LTC	Facilitator
			Team members coordinate care between each other	Facilitator
			Interdisciplinary or entire team used to develop care plans	Facilitator
			LTC have committees or groups that help to provide the best evidence to inform care	Facilitator
			Education of friend and family caregivers enables better agitation and/or aggression care among people with dementia in LTC	Facilitator
			Personalized and interdisciplinary approaches to care improve confidence in care plans	Facilitator
			Not all LTC sites have access to necessary interdisciplinary team members	Barrier
			Staff from different cultural backgrounds respond differently to agitation	Barrier
			Different healthcare providers perceive planning of care to be specific to their professional roles	Barrier
			Lack of Communication between LTC centres	Barrier
		Beliefs about capabilities	Lack of action among care workers	Barrier
			Lack of competency of staff to deliver care	Barrier
			Lack of Coordination of Care among team members in LTC	Barrier
			Staff only have personal background knowledge as their training, with no extra education from long-term care	Barrier
			Lack of Inclusion of Needs and Values of family and residents	Barrier
			Inconsistent training for health care providers caring for people with dementia with agitation	Barrier
			Lack of training for healthcare providers caring for agitation or aggression among people with dementia	Barrier
			Using family members to help provide care	Facilitator
			Adequate training is provided to increase competency and expertise among LTC staff	Facilitator
			Education of friend and family caregivers enables better agitation and/or aggression care among people with dementia in LTC	Facilitator
			Education among healthcare providers for management approaches for agitation and/or aggression enables better care	Facilitator
			Confidence in Care Plan	Facilitator
			The focus of the care plan needs to align with goals of care for the resident	Facilitator
			Not all LTC sites have access to necessary interdisciplinary team members	Barrier
		Optimism	Confidence in Care Plan	Facilitator
			The focus of the care plan needs to align with goals of care for the resident	Facilitator
		Beliefs about Consequences	Lack of Inclusion of Needs and Values of family and residents	Barrier
			Too many staff members handling a patient resulting in agitation	Barrier
			Confidence in Care Plan	Facilitator
		Intentions	Caregivers may not always understand how agitation and/or aggression impacts patient care	Barrier
			Caregivers may under-report symptoms	Barrier
			Lack of attention to hypoactive behaviours	Barrier
			Lack of follow-up of patient agitation symptoms	Barrier
			Healthcare providers are not communicating care practices with residents	Barrier
			We can identify an unmet need, but there can be difficulty with solving it	Barrier
			Changing language around agitated and aggressive behaviours to be more patient-centred	Facilitator
			Hiring someone to carry out interventions or day to day affairs with resident	Facilitator
			Healthcare providers need to be able to self-regulate when providing care to aggressive residents	Facilitator
			Using family members to help provide care	Facilitator
			Team members coordinate care between each other	Facilitator
			Hiring recreational therapist privately for residents with agitation	Facilitator
			Personalized and interdisciplinary approaches to care improve confidence in care plans	Facilitator
			The focus of the care plan needs to align with goals of care for the resident	Facilitator
			Having a checklist of precipitants to consider (e.g. basic needs: food, drink, pain, medication, etc.) aids in management plans	Facilitator
			Care plans for all interventions need to be tailored and patient-centred	Facilitator
			Ensuring that the follow-up of agitated symptoms is integrated into care practices	Facilitator
		Goals	Caregivers may not always understand how agitation and/or aggression impacts patient care	Barrier
			Caregivers may under-report symptoms	Barrier
			Lack of follow-up of patient agitation symptoms	Barrier
			Healthcare providers are not communicating care practices with residents	Barrier
			Lack of communication between health facilities	Barrier
			We can identify an unmet need, but there can be difficulty with solving it	Barrier
			Changing language around agitated and aggressive behaviours to be more patient-centred	Facilitator
			Hiring someone to carry out interventions or day to day affairs with resident	Facilitator
			Using family members to help provide care	Facilitator
			Team members coordinate care between each other	Facilitator
			Interdisciplinary or entire team used to develop care plans	Facilitator
			Hiring recreational therapist privately for residents with agitation	Facilitator
			Confidence in Care Plan	Facilitator
			The focus of the care plan needs to align with goals of care for the resident	Facilitator
			Ensuring that the follow-up of agitated symptoms is integrated into care practices	Facilitator
			Care plans for all interventions need to be tailored and patient-centred	Facilitator
			Lack of Communication between LTC centres	Barrier
	Automatic	Reinforcement	Lack of Coordination of Care among team members in LTC	Barrier
			Lack of follow-up of patient agitation symptoms	Barrier
			Communication with staff is effective among persons involved with the care or planning of care in LTC	Facilitator
			Team members coordinate care between each other	Facilitator
			Interdisciplinary or entire team used to develop care plans	Facilitator
			Personalized and interdisciplinary approaches to care improve confidence in care plans	Facilitator
			Care plans for all interventions need to be tailored and patient-centred	Facilitator
			Ensuring that the follow-up of agitated symptoms is integrated into care practices	Facilitator
		Emotion	Decline in mental status or increased dementia impeding care for agitation	Barrier
			Difficulties among LTC staff to understand the residents’ needs (e.g. likes, dislikes)	Barrier
			Resident personal qualities as barrier to care	Barrier
			Residents having difficulty communicating needs	Barrier
			It is important to look for triggers, contributing causes, and unmet needs that lead to agitation and/or aggression	Barrier
			Having a checklist of precipitants to consider (e.g. basic needs: food, drink, pain, medication, etc.) aids in management plans	Facilitator
			Residents respond better to some staff members and disciplines than others (e.g. rec therapy)	Facilitator

**Table 4:** Codes Related to Acute/Severe and Mild/Moderate Agitation and/or Aggression Treatment, mapped to the TDF and the COM-B Model. Codes related to acute/severe agitation and/or aggression are written in red, whilst those related to mild/moderate treatment are written in black

COM-B		TDF Domain	Codes	Barrier or Facilitator
Capability	Psychological	Knowledge	Interactions with disease, drugs and foods can be barriers to using medication (biological mechanisms)	Barrier
			Severity of agitation can be a barrier to the use of some medications	Barrier
			Lack of education among friend and family caregivers on drug approaches for agitation and aggression	Barrier
			Lack of non-pharmacological interventions available for agitation or aggression	Barrier
			Lack of training specifically for non-pharmacological treatment approaches among LTC staff	Barrier
			Needing to use trial and error to choose non-pharmacological approach	Barrier
			Gentle Persuasion Approach taught among staff	Facilitator
			Specifically assessing basic needs as first line non-pharmacological treatment	Facilitator
			Ensuring staff have the competence and training to administer non-pharmacological treatment approaches	Facilitator
			Non-pharmacological interventions are only administered by nursing staff, not physicians, thus barriers to use are not known by physicians	Barrier
			Best treatment approach is dependent on the person (drug vs. non-drug)	Facilitator
		Cognitive and Interpersonal skills	Agitation symptoms are too severe (e.g. safety concerns) limiting non-pharmacological interventions but permitting pharmacological interventions	Barrier/ Facilitator
			Lack of non-pharmacological interventions available for agitation or aggression	Barrier
			Lack of training specifically for non-pharmacological treatment approaches among LTC staff	Barrier
			Needing to use trial and error to choose non-pharmacological approach choose non-pharmacological approach	Barrier
			Gentle Persuasion Approach taught among staff	Facilitator
			Specifically assessing basic needs as first line non-pharmacological treatment	Facilitator
			Ensuring staff have the competence and training to administer non-pharmacological treatment approaches	Facilitator
			Treatment for agitation depends on the confidence and education of staff to administer non-pharmacological interventions	Facilitator
			Use of medication because it helps address agitated behaviours related to dementia	Facilitator
			Staff are afraid to use non-pharmacological interventions	Barrier
			IM administration route eases ability to administer medication	Facilitator
			Best treatment approach is dependent on the person (drug vs. non-drug)	Facilitator
		Memory, Attention and Decision Making Processes	Comorbid neuropsychiatric diagnosis can conflict with treating agitation symptoms	Barrier
			Difficulty coordinating timing for intervention among a group of residents (E.g. reluctance to participate in non-pharmacological activities)	Barrier
			Advancement in dementia results in frequent changes in non-pharmacological treatment plan needed	Barrier
			Loss of personal traits or skills after administering medication for agitation	Barrier
		Behavioural Regulation	Overuse of restraints	Barrier
			Having non-pharmacological options available such as verbal de-escalation, wait and re-approach, and redirection can be critical for acute or severe agitation	Facilitator
			Agitation symptoms are too severe (e.g. safety concerns) limiting non-pharmacological interventions but permitting pharmacological interventions	Barrier/ Facilitator
			Using chemical restraints (i.e. medications) because agitation symptoms do not respond to other interventions	Facilitator
			Acute/severe agitation can warrant emergency services	Barrier
			Challenges in physically administering medication (e.g.; medication administration can be traumatizing for a person with dementia)	Barrier
			Comorbid neuropsychiatric diagnosis can conflict with treating agitation symptoms	Barrier
			Poor response or worsening of behaviour when medications were used	Barrier
			Reliance on medications	Barrier
			Adverse side effects of medications	Barrier
			Use of Medication because it is convenient	Barrier
			Not all types of agitation are responsive to medications	Barrier
			Routine monitoring of non-pharmacological approaches	Facilitator
			Routine monitoring of medications	Facilitator
			Positive outcomes from non-pharmacological treatments for agitation	Facilitator
			Use of medication because it helps address agitated behaviours related to dementia	Facilitator
			IM administration route eases ability to administer medication	Facilitator
			Using documentation to monitor interventions	Facilitator
			Some residents do respond well to medications for agitation and/or aggression	Facilitator
			Ensuring plans are in place to reassess residents to potentially deprescribe medication	Facilitator
	Physical	Physical Skills	-	-
Opportunity	Social	Social influences	Drug shortages and availability can be a barrier to the use of some medications	Barrier
			Challenges in identifying side effects from the drugs	Barrier
			Challenges in monitoring medications (i.e. no monitoring of medications)	Barrier
			Lack of non-pharmacological interventions available for agitation or aggression	Barrier
			Difficulty coordinating timing for intervention among a group of residents (E.g. reluctance to participate in non-pharmacological activities)	Barrier
			Advancement in dementia results in frequent changes in non-pharmacological treatment plan needed	Barrier
			Easy to access prescriptions for agitation medications	Barrier/Facilitator
			Use of Medication Because it is convenient	Barrier
			Staff pressures on physicians to move to medication sooner	Barrier
			Resources are available that support the use of non-pharmacological interventions (e.g. geriatric mental health)	Facilitator
	Physical	Environmental Context and Resources	Drug shortages and availability can be a barrier to the use of some medications	Barrier
			Challenges in identifying side effects from the drugs	Barrier
			Challenges in monitoring medications (i.e. no monitoring of medications)	Barrier
			Challenges in physically administering medication (e.g.; medication administration can be traumatizing for a person with dementia)	Barrier
			Lack of non-pharmacological interventions available for agitation or aggression	Barrier
			Difficulty coordinating timing for intervention among a group of residents (E.g. reluctance to participate in non-pharmacological activities)	Barrier
			Advancement in dementia results in frequent changes in non-pharmacological treatment plan needed	Barrier
			Use of Medication because it is convenient	Barrier
			Not all types of agitation are responsive to medications	Barrier
			Easy to access prescriptions for agitation medications	Barrier/Facilitator
			Lack of sensory experience non-pharmacological approaches	Barrier
			Intentional use of non-pharmacological treatment strategies	Facilitator
			No regular guidelines to use restraints for agitated patients	Facilitator
			Takes time to acquire consent for a mechanical restraint	Barrier
			Staff pressures on physicians to move to medication sooner	Barrier
			Resources are available that support the use of non-pharmacological interventions (e.g. geriatric mental health)	Facilitator
Motivation	Reflective	Social/Professional Role and Identity	Lack of education among friend and family caregivers on drug approaches for agitation and aggression	Barrier
			Having familiar and developing trust with healthcare providers each time to administer non-pharmacological support for residents	Facilitator
			Although doctors prescribe, the whole interdisciplinary team reports on the effectiveness of treatments	Facilitator
			Takes time to acquire consent for a mechanical restraint	Barrier
			Staff are afraid to use non-pharmacological interventions	Barrier
			Non-pharmacological interventions are only administered by nursing staff, not physicians, thus barriers to use are not known by physicians	Barrier
			Staff pressures on physicians to move to medication sooner	Barrier
			Families or caregivers may not want medications used for the resident	Barrier
		Beliefs about capabilities	Interactions with disease, drugs and foods can be barriers to using medication (biological mechanisms)	Barrier
			Severity of agitation can be a barrier to the use of some medications	Barrier
			Challenges in identifying side effects from the drugs	Barrier
			Challenges in monitoring medications (i.e. no monitoring of medications)	Barrier
			Choosing non-pharmacological approaches as first line	Facilitator
			Ensuring staff have the competence and training to administer non-pharmacological treatment approaches	Facilitator
			Staff are afraid to use non-pharmacological interventions	Barrier
		Optimism	Seeing the patient improve with medication (E.g. making patients more content)	Facilitator
			Positive outcomes from non-pharmacological treatments for agitation	Facilitator
			Choosing non-pharmacological approaches as first line	Facilitator
			Treatment for agitation depends on the confidence and education of staff to administer non-pharmacological interventions	Facilitator
			Some residents do respond well to medications for agitation and/or aggression	Facilitator
		Beliefs about Consequences	Agitation symptoms are too severe (e.g. safety concerns) limiting non-pharmacological interventions but permitting pharmacological interventions	Barrier/Facilitator
			Using chemical restraints (i.e. medications) because agitation symptoms do not respond to other interventions	Facilitator
			Acute/severe agitation can warrant emergency services	Barrier
			Poor response or worsening of behaviour when medications were used	Barrier
			Reliance on medications	Barrier
			Risk of using non-pharmacological approach (e.g. behaviour does not improve)	Barrier
			Adverse side effects of medications	Barrier
			Needing to use trial and error to choose non-pharmacological approach	Barrier
			Seeing the patient improve with medication (E.g. making patients more content)	Facilitator
			Positive outcomes from non-pharmacological treatments for agitation	Facilitator
			Choosing non-pharmacological approaches as first line	Facilitator
			Specifically assessing basic needs as first line non-pharmacological treatment	Facilitator
			Use of medication because it helps address agitated behaviours related to dementia	Facilitator
			Inconsistent monitoring of interventions	Barrier
			Ensuring plans are in place to reassess residents to potentially deprescribe medication	Facilitator
		Intentions	Agitation symptoms are too severe (e.g. safety concerns) limiting non-pharmacological interventions but permitting pharmacological interventions	Barrier/Facilitator
			Using chemical restraints (i.e. medications) because agitation symptoms do not respond to other interventions	Facilitator
			Acute/severe agitation can warrant emergency services	Barrier
			Needing to use trial and error to choose non-pharmacological approach	Barrier
			Lack of sensory experience non-pharmacological approaches	Barrier
			Routine monitoring of non-pharmacological approaches	Facilitator
			Routine monitoring of medications	Facilitator
			Seeing the patient improve with medication (E.g. making patients more content)	Facilitator
			Specifically assessing basic needs as first line non-pharmacological treatment	Facilitator
			No regular guidelines to use restraints for agitated patients	Facilitator
			IM administration route eases ability to administer medication	Facilitator
			Some residents do respond well to medications for agitation and/or aggression	Facilitator
			Challenges in monitoring medications (i.e. no monitoring of medications)	Barrier
			Best treatment approach is dependent on the person (drug vs. non-drug)	Facilitator
		Goals	Agitation symptoms are too severe (e.g. safety concerns) limiting non-pharmacological interventions but permitting pharmacological interventions	Barrier/Facilitator
			Using chemical restraints (i.e. medications) because agitation symptoms do not respond to other interventions	Facilitator
			Needing to use trial and error to choose non-pharmacological approach	Barrier
			Lack of sensory experience non-pharmacological approaches	Barrier
			Routine monitoring of non-pharmacological approaches	Facilitator
			Routine monitoring of medications	Facilitator
			Seeing the patient improve with medication (E.g. making patients more content)	Facilitator
			Specifically assessing basic needs as first line non-pharmacological treatment	Facilitator
			No regular guidelines to use restraints for agitated patients	Facilitator
			IM administration route eases ability to administer medication	Facilitator
			Best treatment approach is dependent on the person (drug vs. non-drug)	Facilitator
	Automatic	Reinforcement	Overuse of restraints	Barrier
			Having non-pharmacological options available such as verbal de-escalation, wait and re-approach, and redirection can be critical for acute or severe agitation	Facilitator
			Routine monitoring of non-pharmacological approaches	Facilitator
			Routine monitoring of medications	Facilitator
			Having familiar and developing trust with healthcare providers each time to administer non-pharmacological support for residents	Facilitator
			Inconsistent monitoring of interventions	Barrier
			Using documentation to monitor interventions	Facilitator
			Challenges in monitoring medications (i.e. no monitoring of medications)	Barrier
			Ensuring plans are in place to reassess residents to potentially deprescribe medication	Facilitator
		Emotion	Challenges in physically administering medication (e.g.,; medication administration can be traumatizing for a person with dementia)	Barrier
			Comorbid neuropsychiatric diagnosis can conflict with treating agitation symptoms	Barrier
			Loss of personal traits or skills after administering medication for agitation	Barrier
			Not all types of agitation are responsive to medications	Barrier

### Barriers and facilitators to care at detection and diagnosis of agitation and/or aggression

Several main facilitators were described at detection and diagnosis. Agitation diagnostic tools were reported as advantageous because they can be *easily administered by different healthcare professionals and produce easy-to-understand results*. As well, using agitation diagnostic tests were considered useful because they allow healthcare practitioners to *compare agitation between residents* and *keep assessments objective*. Interview participants also advocated for increased *training among healthcare providers to use agitation screening tools.* Lastly, allied healthcare workers praised using the *Resident Assessment Instrument (RAI)* along with *counting the number of aggressive or agitated incidents* as facilitators to diagnose agitation.

*“Well, the advantage is, it actually outlines the signs and symptoms […] so that it's readily available and reproducible […] and somebody who's unskilled can actually use a lot of these tools.”* (Participant 3)

Several barriers to care at detection and diagnosis were identified. Firstly, certain *diagnostic tests may prove difficult to administer* because they *are not adapted for persons with cognitive impairment.* Interviewees reported *difficulties in understanding how agitation diagnostic tests work.* And, differing levels of *healthcare provider familiarity with agitation diagnostic tools* may affect how comfortable and competent they are with administering them. There were logistical challenges to using agitation tools because tools were commonly *time consuming*, and required adequate *healthcare provider availability*. As well, *diagnosis of cognitive issues* took a long time, which *delays diagnosis of agitation and/or aggression:*

“*[T]he whole process of diagnosis took about three years, and the cognitive neurologist was seeing us every six months, and she would test him every time with different mental tests…*” (Participant 1)

Furthermore, *diagnosis of agitation and/or aggression took a long time,* which can delay the onset of treatment. Another caregiver described a *lack of available diagnostic tests for agitation* for PLWD in LTC. Diagnostic care practices also commonly *overlook hypoactive behaviours in dementia* that are comorbid to agitation and/or aggression:

“*The hyperactive [resident] usually attracts the attention of everybody because they're distressed, yelling, screaming, fidgeting, wandering, moving, so they're active, whereas the hypoactive – that's where people can be missed*” (Participant 3).

Although cognitive impairment and hypoactive behaviours are not specific to agitation and/or aggression, a delay in diagnosis of cognitive impairment was interpreted by participants to consequently delay the detection of associated agitated and/or aggressive behaviours.

### Barriers and facilitators to coordination and management of care of agitation and/or aggression

A key facilitator to the coordination and management of care was *using family members to help provide care*, to help calm residents and direct the course of care. Secondly, interviewees supported *using personalized and interdisciplinary approaches to care* to *improve confidence in care plans*. Components of personalized care included having a *supportive and personalized environment* for the resident to physically live, and *having a checklist of precipitants to consider (e.g., basic needs, food, *etc*.)* for each resident. As well, *specialized or interdisciplinary care teams* were needed to develop care plans and management strategies:

“*[W]e do have our interdisciplinary team that regularly debates and we discuss each resident several times a year, and then more so if needs arise. And so it's anywhere from HCA to physio, TRT, social work, dietary, the entire interdisciplinary team.*” (Participant 10)

In terms of barriers, several participants reported a *lack of action among care workers* to address agitation and/or aggression concerns among residents, and a *lack of staff-to-staff and staff-to-family caregiver communication* as a barrier to consistent and quality care for agitation. There were *cultural and language barriers to care* for residents identifying as persons of colour, and *constantly changing directives in LTC facilities* or a *lack of existing directives* to address agitation and/or aggression. Environmental barriers included the presence of constant *loud noises* and *unideal room configurations* for PLWD in LTC. Finally, *a lack of available resources* to provide care was raised as a crucial barrier to care, with a particular focus on the *cost of care*, *staffing issues* and *limited time for healthcare providers to provide care*.

“*So there was one LPN* [licensed practical nurse]*, and three healthcare aides for 30 patients with dementia. It wasn't enough.*” (Participant 1)

### Barriers and facilitators to treatment for mild/moderate agitation and/or aggression

There were several reported facilitators to administering medications including *routine monitoring of medications*, having an *interdisciplinary team available to prescribe medications*, and an *easy access to prescriptions for agitation medications:*

“*And so how [medications are] actually prescribed is, it becomes the doctor's orders, ultimately, but the doctor does rely on feedback from the nursing staff as well on what's been effective or not*.” (Participant 9)

Various barriers to using medication to treat mild-to-moderate agitation and/or aggression included *barriers due to biological mechanisms*, presentation of *severe agitation,* and *drug shortages and availability*. There were also *challenges in identifying side effects from the drugs*, *in monitoring the medications*, and *in physically administering medication* to residents:

“*Challenges in administration. Challenges if there is not enough monitoring to see the effects of these drugs. Challenges in explaining to the caregivers what to look for in terms of side effects or other effects from the drugs.*” (Participant 6)

Facilitators to using non-pharmacological interventions included incorporating *intentional use of non-pharmacological treatment strategies, routine monitoring of non-pharmacological approaches*, and *having familiar and trustable healthcare providers* with the *competence and training to administer non-pharmacological treatment approaches:*

*“They use different activities - recreational activities. […] So they would try to redirect him with activities.”* (Participant 1)

In terms of barriers to using non-pharmacological interventions for agitation and/or aggression, interviewees reported a *lack of training specifically for non-pharmacological treatment approaches* among healthcare providers, and a *lack of non-pharmacological interventions available* in LTC. A logistical challenge included *difficulty coordinating timing for interventions among groups of residents.* Treatment strategies often *relied on medication because it is convenient*, with an *easy access to prescriptions* for agitation medications, thus non-pharmacological interventions were underused. The need to *use trial and error* to select a non-pharmacological intervention was also inconvenient.

“*I think the only thing is that [non-pharmacological treatments are] actually not used [that] often. The default is drugs, […] because drugs are the easiest. Given the staffing shortage, it seems to be the default.*” (Participant 6)

### Barriers and facilitators to treatment for acute/severe agitation and/or aggression

A key facilitator to non-pharmacological treatment for acute/severe agitation and/or aggression was having *non-pharmacological options available* for acute/severe agitation and *having a least restraint policy* in LTC*.* A facilitator to pharmacological treatment was choosing to *use chemical restraints because agitation and/or aggression symptoms are too severe* due to safety concerns for the resident and healthcare providers:

“*We need something to work quickly because somebody else will get hurt if we don't act sooner.*” (Participant 8)

An overall barrier for acute/severe agitation treatment was the *reliance on physical and/or chemical restraints*. As well, *agitation symptoms being too severe* served as a barrier to using non-pharmacological interventions for acute/severe agitation and/or aggression:

“*When a person is in that extreme agitation [...] you've determined that this is the immediate course of action [...] to get Haldol [or] Seroquel, whatever, into that person.*” (Participant 10)

Several codes arose regarding barriers and/or facilitators to care at a systemic and policy level in LTC. An *unclear awareness or availability of geriatric medicine or geriatric psychiatry services* in LTC served as a barrier at the detection and diagnosis of agitation and/or aggression. Conversly, having *physicians more actively involved in care* in LTC centres resulted in *less referrals* and was a facilitator to care at detection and diagnosis. Lastly, as previously mentioned, interviewees reported that *having a least restraint policy* in LTC was a facilitator to providing non-pharmacological interventions.

## Discussion

This study identifies key barriers and facilitators to care behaviours for agitation and/or aggression among PLWD in LTC, across 4 major categories: (1) Detection and Diagnosis, (2) Care Coordination and Management, (3) Treatment for mild-to-moderate agitation and, (4) Treatment for acute/severe agitation. Key barriers across the spectrum of care included a limited number of agitation and/or aggression diagnostic measures, a lack of training for managing agitation and/or aggression in LTC, an overuse of physical and chemical restraints among acutely/severely agitated and/or aggressive residents, and an underuse of non-pharmacological interventions. Facilitators included using an interdisciplinary team to deliver care and having competent and trained healthcare providers to administer non-pharmacological interventions. Ultimately, these results advance the care for PLWD in LTC by highlighting key issues needing to be addressed. The findings will support future implementation research endeavours to combat these barriers through targeted interventions to improve the quality of care across Canada.

## Detection and diagnosis

### Specific tools used to detect and diagnose agitation and/or aggression among PLWD in LTC

The most frequently reported methods of diagnosing and monitoring agitation and/or aggression symptoms in LTC centres was through two main charting means: the Behaviour and Symptom Mapping Tools and the RAI (RAI-Minimum Data Set (MDS) 2.0). Interestingly, no interviewee mentioned the use of an agitation and/or aggression psychometric tool, bringing the availability of agitation and/or aggression diagnostic tools in LTC into question. This barrier relates to issues with availability of resources in LTC. Most of the psychometric tools examined in a recent systematic review were not compared to a reference standard, and there were no studies that examined the BSMT or RAI-MDS 2.0 questions [25]. Therefore, there are no reported sensitivity, specificity, or minimally clinical important difference measures seen for these tools. In turn, it is unclear how these tools perform clinically. There are many reasons for this – agitation and aggression are very prominent observable symptoms, and their reporting needs to be tied to antecedent events through informant accounts to be useful to healthcare providers [26]. As well, behavioural and psychological symptoms of dementia (BPSD) often overlap, with agitation and aggression often expressed together, resulting in conflation between symptoms [2]. Beyond tools, there are also other comprehensive approaches to assessing agitation and/or aggression described in the literature, such as the “Describe, Investigate, Create, and Evaluate” (DICE) method [27]. These approaches were also not mentioned in the interviews. To ensure residents are receiving the best means of agitation and/or aggression detection and diagnosis, more research is needed to validate current tools among PLWD in LTC, and determine whether psychometric tools should be implemented in regular practice.

### Using an interdisciplinary care team to diagnose agitation and/or aggression among PLWD in LTC

The diagnosis for agitation and/or aggression is typically finalized by physicians in LTC, using aggregated information collected from members of the interdisciplinary care team. The collaborative approach to care, where all interdisciplinary healthcare providers and/or friends and family caregivers have input into resident care plans, is crucial to the diagnosis and management of agitation and/or aggression. This facilitator demonstrates strengths pertaining to reinforcement of practices, healthcare providers’ perceived identity, and creating goals of care. A collaborative, interdisciplinary approach effectively offsets physician time and increases confidence among physicians to make diagnoses [28, 29]. As well, residents receive a comprehensive assessment outside of a physician’s diagnosis, using the maximized complementary strengths of the entire care team [28, 29]. Interdisciplinary care teams uphold person-centred care values, by addressing the unique needs of each resident whilst giving shared decision making to healthcare providers, residents and family and/or friend caregivers [28, 29]. Given the benefits, any chosen method to detect or diagnose symptoms of agitation and/or aggression should account for interdisciplinary teams and family and/or friend caregivers.

In a recent systematic review, the majority of agitation and/or aggression tools lacked a comprehensive, interdisciplinary assessment of residents [25]. The Behavioral Pathology in Alzheimer’s Disease Rating Scale (BEHAVE-AD) and the Neuropsychiatric Inventory (NPI) were the only tools that seemed to account for multiple stakeholder perspectives (i.e., assessing caregiver distress along with resident symptoms). A potential reason for this is that agitation and/or aggression symptoms are predominantly detected via the observation of residents, or through informant reports of the frequency of symptoms, resulting in only observation-based and informant-rated tools available [26]. However, these assessment methods are limiting, where only observable points of contact with the resident can be evaluated [26]. More research is thus needed to determine whether incorporating an interdisciplinary evaluation approach into current assessment methods is more clinically beneficial to residents.

## Care coordination and management

### Lack of training for managing agitation and/or aggression

Family/friend caregivers and allied healthcare workers felt that training in LTC is inconsistent, lacks staff-to-staff and staff-to-family caregiver communication, and does not properly address resident needs. These issues relate to several challenges, including issues with knowledge and skills among healthcare providers, limited resources, and challenges in staff’s perceived identity. Ultimately, training standards within LTC settings vary province-to-province across Canada [30]. Training for crucial healthcare practitioners in LTC (e.g., physicians, nurses) is not standardized, and often does not embrace a geriatric-focused lens [30]. In the analyses, interviewees raised concerns that these variable care protocols for agitation and/or aggression do not meet residents’ needs. The variability seen in training adversely impacts management of agitation and/or aggression among PLWD in LTC. There is a need for standardized practices for addressing agitation and/or aggression symptoms among PLWD in LTC among healthcare practitioners in LTC, to improve the efficiency and quality of care.

## Mild-to-moderate agitation and/or aggression

### Underusage of non-pharmacological interventions:

Non-pharmacological interventions are considered more efficacious than pharmacological for agitation and/or aggression due to less adverse side effects, greater cost efficiency, and because they address underlying resident needs [11, 31]. Despite this knowledge, healthcare providers lacked education and training on how to administer different non-pharmacological interventions, thus serving as a crucial barrier to agitation care. This barrier reflects issues in resources along with knowledge and skills among healthcare providers. One reason for why knowledge and training are lacking is that processes of selecting and administering non-pharmacological interventions are largely unsystematic and reportedly based on trial-and-error [32]. Consequently, due to time constraints, healthcare practitioners interviewed in this study often resided to using pharmacological interventions rather than non-pharmacological, out of convenience. This issue was corroborated by Janzen et al.’s (2013) findings, where unpredictable environmental factors and healthcare provider and/or resident personal traits (i.e. personality) resulted in arbitrary selection of non-pharmacological approaches [9].

Through the discussions, a key theme that emerged was a need for better upstream, person-centred approaches for the prevention of agitation and/or aggression. For example, one participant noted that physicians are active in LTC and respond quickly to behaviours, but a separate participant pointed out that such responses typically resort to using chemical restraints (Additional File 3). This issue highlights how agitation and/or aggression are currently being addressed in a downstream manner, after behaviours have manifested. Ultimately, person-centred approaches to prevent agitation and/or aggression use individual unique characteristics, strengths, and weaknesses to recognize and meet individual unmet needs, thus preventing agitation and/or aggression prior to their onset [33]. A previous meta-analysis demonstrated that using person-centred care interventions significantly reduces agitation amongst other neuropsychiatric symptoms [33]. For example, the “Treatment Routes for Exploring Agitation” (TREA) program, along with other therapeutic recreation programs, provide tailored activities to residents, and have demonstrated a reduction of agitation between 10–14 days following completion of these interventions [33]. Therefore, a greater emphasis on person-centred, upstream interventions is needed in LTC to prevent the onset of agitation and/or aggression among residents.

Another issue brought up by family and/or friend caregivers, was the limited number of available non-pharmacological interventions in LTC. Non-pharmacological interventions follow a person-centred approach to address unique behavioural needs of each resident [34]. However, to tailor approaches to each resident, non-pharmacological interventions require extensive time and staffing resources to implement – both of which are lacking in LTC [9]. Both factors are common barriers to implementing non-pharmacological interventions across a range of behavioural symptoms in LTC [24]. For example, Hussin et al. (2021) noted several barriers to implementing non-pharmacological interventions for BPSD in LTC, including limited staff time and training [35]. Likewise, Oldenburger et al. (2022) reported that, although residents require approximately 4.1 h of care time per day to meet needs, they are only receiving about 2.45 h to 3.73 h of care per day [36]. The onset of COVID-19 has further exacerbated issues in staffing and time to provide care [36]. Due to these constraints, a restricted number of non-pharmacological interventions are offered in LTC, thus negatively impacting the quality of care for residents experiencing a variety of health conditions. Given the widespread negative impacts, upstream implementation research is needed to counteract these time and resource constraints, allowing space for more non-pharmacological intervention strategies in LTC.

## Acute/Severe agitation treatment

### Overuse of physical and chemical restraints for acute/severe agitation and/or aggression

A key barrier at acute/severe treatment for agitation and/or aggression was the reliance on physical and chemical (i.e., fast-acting medications) restraints to contain an acutely agitated and/or aggressive resident. This issue relates to challenges in regulating resident behaviours and reinforcement of practices. Acutely agitated and/or aggressive residents were considered at risk of harming themselves or others, thus as needed antipsychotic medications (e.g., Haldol) and mechanical restraints (e.g., chair with a seatbelt) were used. These measures carry significant risks to residents including a loss of dignity, social isolation, shame, and physical harm [37, 38].

Many LTC institutions across Canada have implemented a “Restraint as a Last Resort” policy, where the least restrictive pharmacological, environmental, mechanical, and physical restraints are administered as a last resort practice [39]. Across provinces, similar policies have been implemented by LTC organizers, including Alberta Health Services, Health Prince Edward Island, and the College of Nurses of Ontario [39–41]. Despite least restraints being a shared goal across Canadian LTC centres, the discussions seemed to highlight an increased use of them among residents. Future studies should evaluate whether current uses of restraints across Canadian LTC centres are appropriate.

Several interviewees highlighted redirection, resident isolation and Gentle Persuasive Approach training. Other non-pharmacological approaches seen in the literature for acute/severe agitation and/or aggression include, but are not limited to, non-coercive verbal de-escalation or self-soothing techniques [42, 43]. However, there are barriers to the use of these interventions.

This study featured a myriad of perspectives from persons of differing roles in LTC (Table 1). Due to these diverse roles, different interviewees focused on different points of discussion. For example, physicians presented a clinical lens during discussions on the detection and diagnosis of agitation and/or aggression, along with corresponding pharmacological interventions. In terms of the latter, physicians spoke to barriers in using pharmacological interventions from the pathophysiological aspect, including drug-drug interactions, and biological mechanisms (Additional File 3). In comparison, nurses and allied healthcare workers focused on challenges in the administration of medications, while family caregivers and spouses focused on education barriers surrounding medication use. Furthermore, allied healthcare workers and nurses provided shared experiences regarding the coordination of care for agitation and/or aggression. In particular, allied healthcare workers (E.g.; occupational therapists, recreational therapists) had notable experience conducting non-pharmacological interventions with residents in LTC, and could speak to the barriers and facilitators they had encountered. Lastly, caregivers and spouses presented ideas throughout their interviews from the residents’ perspectives, with themes surrounding their perceived quality of life in LTC.

Few qualitative studies are currently available on the barriers and facilitators to neuropsychiatric care among Canadian LTC centres. Current qualitative literature identifies barriers and facilitators to small-scale implementations in Canadian LTC centres, such as the PIECES education framework [44], but broad-scale qualitative behavioural research has not been conducted. One systematic review exists on the barriers and facilitators to complex interventions for PLWD in LTC, but this study does not focus on widescale barriers to neuropsychiatric care in LTC, and only features 2 studies with a Canadian setting [45]. Taken together, this gap in research can have negative clinical implications, as key barriers to care in Canadian LTC centres are missed. This study thus serves as a crucial step in improving understanding of agitation and/or aggression care in LTC, accounting for a broad range of lived experiences and perspectives.

At a broader context, several findings consistent with studies conducted at a global scale were acquired. For example, interviewees detailed cost barriers, disproportionate staff-to-resident ratios, and limited time to provide care as barriers to coordinating and managing care in Albertan LTC facilities. These findings were also reported by Janzen et al. (2013) and McArthur et al. (2021), where limited time to deliver care and inadequate staffing were also systematic and pervasive issues [9, 30]. Similarly, environmental barriers to agitation care were found, including loud noises and unideal room configurations. This finding is corroborated by Cohen-Mansfield et al.’s (2012) study, where environmental conditions also served as barriers to administering non-pharmacological interventions for a range of behavioral symptoms [24]. Taken together, each of these barriers have served as perpetual challenges over the last decade in diverse LTC settings across North America. These findings thus demonstrate the need for a substantial global knowledge-to-action plan to address these pervasive challenges.

### Limitations and Generalizability

There were several limitations in this study. Despite aiming to interview participants from a broad array of backgrounds and disciplines, the majority (83.3%) of participants identified as White. The lack of diversity in our sample may not reflect the perspectives of persons of colour working or engaging in LTC. Likewise, cultural or spiritual barriers and/or facilitators may have been missed, that more often impact racial minorities across Canada. This bias could potentially impact the generalizability of our results to racialized Canadian communities [e.g., Indigenous, Black, Indigenous, Persons of Colour, etc.].

### Future directions

Several key barriers and facilitators to care for agitation and/or aggression among PLWD in LTC facilities were identified, at detection/diagnosis, care coordination/management, and mild-to-moderate and acute/severe treatment. Given that these barriers were mapped to the TDF, future research efforts can form a substantial knowledge-to-action plan by mapping these TDF domains to the COM-B and subsequently the Behaviour Change Wheel. Therefore, appropriate implementation strategies can be created to change behaviours in LTC to eliminate these barriers to care.

## Conclusions

This qualitative study used semi-structured interviews to identify the main barriers and facilitators to care for agitation and/or aggression among PLWD in LTC found that key barriers included a lack of validated tools to detect agitation and/or aggression, inconsistent and variable training practices among healthcare providers, and a limited number of non-pharmacological interventions available in LTC. Key facilitators were using an interdisciplinary team approach and having competent and trained healthcare providers to administer non-pharmacological interventions. Future research should look towards creating feasible implementation strategies to eliminate the identified barriers, in order to improve care outcomes among PLWD in LTC.

### Supplementary Information

**Supplementary Material 1. **

**Supplementary Material 2. **

**Supplementary Material 3. **

## Data Availability

The dataset generated and analyzed are not publicly available as individual participant interview transcripts cannot be shared beyond the research team listed on the ethics agreement held with the University of Calgary’s Conjoint Health Research Ethics Board. Thematically analyzed data and associated participant quotes are available in Additional File 3. For data requests or inquiries please contact Dr. Zahra Goodarzi at zahra.goodarzi@albertahealthservices.ca.

## Abbreviations

BEHAVE-AD: Behavioral Pathology in Alzheimer’s Disease Rating Scale
LTC: Long-term care
MDS: Minimum Data Set
NPI: Neuropsychiatric Inventory
PLWD: Persons living with dementia
RAI: Resident Assessment Instrument
TDF: Theoretical Domains Framework
