# Supplementary material for: Barriers and facilitators to care for agitation and/or aggression among persons living with dementia in long-term care
Source: BMC Geriatr. 2024 Apr 11;24:330. doi: 10.1186/s12877-024-04919-0 (PMC11008022; doi:10.1186/s12877-024-04919-0)
Supplement: Supplementary file 1 — Supplementary Material 1. [file 12877_2024_4919_MOESM1_ESM.docx]

Additional File 1: Reflexivity Statement

We acknowledge that the extraction of themes and patterns from the qualitative data is influenced by the lived experiences of the researchers involved with this project. The primary analyst is a Master of Science student in Community Health Sciences at the University of Calgary, with introductory experience researching in a LTC setting. The secondary analyst is a geriatrician with extensive experience treating older adult Canadians in clinical settings, and conducting research in the field of gerontology. This study reports all findings in adherence with the COREQ checklist.
