# Supplementary material for: Barriers and facilitators to care for agitation and/or aggression among persons living with dementia in long-term care
Source: BMC Geriatr. 2024 Apr 11;24:330. doi: 10.1186/s12877-024-04919-0 (PMC11008022; doi:10.1186/s12877-024-04919-0)
Supplement: Supplementary file 2 — Supplementary Material 2. [file 12877_2024_4919_MOESM2_ESM.docx]

Additional File 2

Table of Contents

[Additional File 2.1: Interview Guide for Healthcare Providers 1](#_Toc148527337)

[Additional File 2.2: Interview Guide for Residents and Family and/or Friend Caregivers: 5](#_Toc148527338)

# **Additional File 2.1: Interview Guide for Healthcare Providers**

**Part I: Preamble For interviews for Health Care Providers**

*Interviews are to be completed with nurses, physicians, and allied health professionals. This is the generic interview guide to which minor adjustments will be made to tailor to individuals from each of these three groups.*

Hello, my name is [*researcher name*] and I am a [*student, research assistant, researcher]* from the Department of [*Medicine/Community Health Sciences/Neuroscience*] at the University of Calgary. I’m calling in regard to the study “Determining Barriers and Facilitators to Care for Agitation and/or Aggression among Persons Living with Dementia in Long-term Care”. As part of this study, we would like to get your insight your general experience on the process of detecting, diagnosing, managing and treating agitation and/or aggression symptoms among persons with dementia in long-term care. The interview will take approximately 45- 60 minutes. We will do our best to complete the interview within this time frame to respect your time.

All information you share in this interview will be confidential and your answers will not be shared with your employer. Your participation will not affect your employment. Although I will be recording the interview using an audio recorder to help me transcribe the conversation, any data that identifies participants (such as names and locations) will be removed from the transcript to protect anonymity.

Once the interview has been completed, the audio file will be uploaded onto a password-protected, encrypted University of Calgary computer and the audio file will be deleted from the recording device. The audio recording will be accessible only to study personnel listed on the ethics agreement and the audio file will be transcribed by a third party who will sign a confidentiality agreement, and will otherwise not be shared with anyone outside of myself and members of the study team. Storage of the audio file will adhere to the University of Calgary’s storage and data retention guidelines. If during the course of our interview you identify that someone is at risk of harm, then I will be required to notify the study leaders and may be required to release this information to appropriate authorities.

Participation in this study is voluntary. You may skip any questions that you do not wish to answer, and you may withdraw from this study up to the point when the data has been anonymized and aggregated and the analysis has been completed. After this time, you will not be able to remove your data from the study. Should you withdraw from the study after your interview has been completed, you may elect to have your data removed or included in the study. We can end the interview at any point, or take a break if needed.

**Make sure participant has completed the informed consent process*

**Ask participant if everything stated is clear*

**Ask participant if you may begin recording*

The following questions are related to your experience as a care provider in the LTC setting. You may answer in any way you wish, recalling that your responses will be anonymized, and confidentiality respected.

| **Interview Guide** | | |
| --- | --- | --- |
| **Topics** | **Questions and Related Prompts/Probes** | **TDF Domain Covered** |
| Detection and Observation of Agitation and/or Aggression Among PLWD in LTC | 1. Please briefly describe your current role working in LTC? 2. Can you describe the common behaviours you see when a PLWD is experiencing agitation and/or aggression in LTC? 3. What tools or assessment scales are used in clinical environments to detect the presence OR severity of agitation and/or aggression among PLWD? 4. In your experience, what are the advantages/disadvantages to using a particular tool/measure(s) to detect agitation and/or aggression among PLWD? 5. How are agitation and/or aggression symptoms monitored and documented over time by healthcare practitioners? | - - - 1. Social/professional role and identity       2. Knowledge       3. Knowledge, Skills, Reinforcement, Memory attention and decision processes       4. Skills, Reinforcement, Beliefs about capabilities, Optimism       5. Skills, Goals, Intentions, Reinforcement |
| Diagnosis of Agitation and/or Aggression Among PLWD in LTC | 1. How is a diagnosis of agitation and/or aggression made?    1. How is cognitive status assessed among agitated residents? 2. When considering a diagnosis of agitation and/or aggression, what common factors (e.g. needs, precipitants, or perpetuating), are assessed? | - - - 1. Knowledge, Skills       2. Knowledge, Skills |
| Management of Care | 1. Who contributes to developing treatment plans to address contributors to agitation and/or aggression symptoms? 2. How are residents, family and friend caregiver preferences and values included in the diagnosis and management of agitation and/or aggression? 3. What healthcare practitioners are involved in the management of care for agitation and/or aggression among the PLWD in LTC? (e.g. clinical nurse specialists, occupational therapists, recreational therapists, behavioral support specialists) 4. Is the care home serviced by geriatric medicine and/or geriatric psychiatry?    1. If yes, when/at what point would you call geriatric psychiatry or a geriatrician to examine the resident? | - - - 1. Social/Professional Role and Identity       2. Social/Professional Role and Identity, Beliefs about capabilities, Social influences       3. Social/professional Role and Identity, Beliefs about capabilities       4. Social/professional role and identity |
| Treatment for Agitation/Aggression | 1. What non-pharmacological interventions for agitation and/or aggression are used among persons living with dementia? 2. How do you or providers choose between non-pharmacological interventions?    1. Which are tried first?    2. Do you feel comfortable using a non-pharmacological intervention to manage agitation and/or aggression symptoms in persons living with dementia? 3. What are some of the barriers or potential difficulties that you perceive to administering non-pharmacological interventions to manage agitation and/or aggression symptoms in PLWD? 4. Are there any benefits to recommending a non-pharmacological intervention over a pharmacological one to treat agitation and/or aggression symptoms among PLWD?    1. Are there any risks to the use of the non-pharmacological therapies? 5. How are pharmacological (drug) interventions selected to manage agitation and/or aggression symptoms?    1. What are some reasons why pharmacological intervention may be preferred over the other? 6. What are some of the barriers or potential difficulties with administering pharmacological interventions to manage agitation and/or aggression symptoms in PLWD? 7. How are risks and benefits weighed when administering pharmacological interventions? 8. What interventions are used for acute/severe agitation symptoms among persons living with dementia?    1. Are there non-pharmacological interventions? 9. In your opinion, are non-pharmacological interventions as efficacious as pharmacological interventions to manage agitation and/or aggression symptoms in PLWD? 10. What resources are available that facilitate the use of pharmacological interventions over non-pharmacological interventions for agitation and/or aggression in PLWD? 11. Are there other external factors that influence your ability or choice to use a course of treatment over another? 12. How is the effectiveness of these interventions followed over time?     1. Are there specific tools used?     2. In your experience, are there advantages or disadvantages to using a particular tool/measure(s) to assess which intervention option to follow? 13. Is there something else that we have not yet discussed today that you feel is important to consider when it comes to the usage of non-pharmacological interventions over pharmacological ones in LTC? | - - - 1. Memory attention and decision making, knowledge       2. Reinforcement, Knowledge, Memory attention and decision making, intentions, goals       3. Optimism, Emotion, beliefs about capabilities, knowledge       4. Optimism       5. Reinforcement, Knowledge, memory attention and decision making, intentions       6. Optimism, Knowledge, Beliefs about capabilities, Emotion       7. Memory attention and decision making, Skills, Knowledge, Intentions, Goals       8. Behavioral regulation, Knowledge, reinforcement       9. Beliefs about capabilities, Optimism, Beliefs about consequences       10. Environmental Context and Resources       11. Social influences       12. Reinforcement, skills, knowledge |

Thank you statement: So, that brings us to the end of this interview. I’d like to thank you for taking the time to talk to us today and providing your insight on this topic. If you have any further questions or comments, please don’t hesitate to contact me or one of the other researchers. Thank you.

# Additional File 2.2: Interview Guide for Residents and Family and/or Friend Caregivers:

**Part I: Preamble For interviews for Persons with Lived experience and Care-Partners**

*Interviews are to be completed with residents and care-partners. This is the generic interview guide to which minor adjustments may be made to explain or tailor questions to the interviewee.*

Hello, my name is [*researcher name*] and I am a [*student, research assistant, researcher]* from the Department of [*Medicine/Community Health Sciences/ Neuroscience*] at the University of Calgary. I’m calling in regard to the study “Determining Barriers and Facilitators to Care for Agitation and/or Aggression among Persons Living with Dementia in Long-term Care”. As part of this study, we would like to get your insight your general experience on the process of detecting, diagnosing, managing and treating agitation and/or aggression symptoms among persons with dementia in long-term care. The interview will take approximately 45- 60 minutes. We will do our best to complete the interview within this time frame to respect your time.

All information you share in this interview will be confidential and your answers will not be shared with your employer. Your participation will not affect your employment. Although I will be recording the interview using an audio recorder to help me transcribe the conversation, any data that identifies participants (such as names and locations) will be removed from the transcript to protect anonymity.

Once the interview has been completed, the audio file will be uploaded onto a password-protected, encrypted University of Calgary computer and the audio file will be deleted from the recording device. The audio recording will be accessible only to study personnel listed on the ethics agreement and the audio file will be transcribed by a third party who will sign a confidentiality agreement, and will otherwise not be shared with anyone outside of myself and members of the study team. Storage of the audio file will adhere to the University of Calgary’s storage and data retention guidelines. If during the course of our interview you identify that someone is at risk of harm, then I will be required to notify the study leaders and may be required to release this information to appropriate authorities.

Participation in this study is voluntary. You do not have to answer questions if they make you uncomfortable and you may withdraw from this study up to the point at which the analysis has been completed. Once the analysis has been completed, you will not be able to remove your data from the study because it will be anonymous and combined with information from other participants. If you choose to withdraw after the interview has been completed, you may elect to have your data removed or included in the study. We can stop the interview at any point or take a break if needed.

**Ask participant if everything stated is clear*

**Make sure participant has completed the informed consent process*

**Ask participant if you may begin recording*

The following questions are related to your experience as a resident or care partner in the LTC setting. You can answer in any way you wish, keeping in mind your responses will be anonymous.

| **Interview Guide** | | |
| --- | --- | --- |
| **Topics** | **Questions and Related Prompts/Probes** | **TDF Domain Covered** |
| Introductory Questions | 1. Please briefly describe your current situation regarding living or having a relative in long term care? 2. Have you or your family or friend been diagnosed with dementia? 3. Has your relative with dementia experienced agitation/aggression? (or if interviewee is the resident: Have you experienced agitation/aggression?) | - - - 1. Environmental context and resources, social/professional role and identity       2. Knowledge       3. Knowledge |
| Detection and Diagnosis of Agitation and/or Aggression | 1. How were these symptoms recognized or diagnosed? 2. Did the healthcare practitioner use any tools or questionnaires? 3. How was the diagnosis of agitation and/or aggression communicated to you? 4. How did you/ the resident feel emotionally about the diagnosis of agitation and/or aggression? 5. How did healthcare workers include your (i.e. caregivers and residents) preferences and values in the diagnosis with respect to agitation and/or aggression?   In the care plan/management? | Knowledge, Skills, Reinforcement, Beliefs about capabilities  Goals, Memory attention and decision processes, Emotion  Emotion, Beliefs about consequences  Social/professional role and identity, Beliefs about capabilities, social influences |
| Management and Treatment of Agitation/Aggression | 1. What interventions were tried to manage aggression/agitation? 2. Goals of care refer to a patient-centric approach to document patient values, wishes, and goals in the context of medically appropriate treatment. For example, a goal of care could be what a patient wants to achieve during a hospital visit. How were the goals of care being addressed by using these interventions? 3. Did the health care provider explore other intervention options to manage aggression/agitation? If so, which ones?    1. How did you feel about implementing the intervention before and after it was included in the care plan? 4. Did the health care provider explain why this intervention was used? Did the health care provider answer all your questions? 5. Who was involved in these discussions? 6. Are you aware of the advantages and disadvantages of this intervention? 7. What challenges do you perceive are associated with the intervention assigned to you/your resident? 8. How did healthcare practitioners address any of the concerns you had regarding the specific non-pharmacological intervention used? 9. Did other health care providers help you understand the intervention used to manage aggression/agitation? If yes, which provider (e.g., nurse, physician etc.)? 10. What education and information did you receive about the intervention used to manage aggression/agitation? 11. What resources and/or aspects of the care environment do you feel helped contributed to supporting the use of a non-pharmacological intervention for the resident? 12. Was a non-pharmacological intervention ever discussed as an option to manage aggression/agitation? A list here of non-pharmacological interventions is provided for reference:  - Social activities - Cognitive Stimulation Therapy - Culturally-appropriate reminiscence therapy - Life review, life reflection, legacy work - Music therapy - Therapeutic Touch - Aromatherapy - Animal Therapy - Horticulture Therapy - Light Therapy - Technology-based Interventions - Recreation Therapy - Exercise  1. If yes to question 12, which intervention(s) were chosen to manage aggression/agitation? 2. If yes to question 12, how did staff show preparedness, understanding, and readiness to administer the non-pharmacological approach? 3. If yes to question 12, why were these intervention(s) effective to manage aggression/agitation? 4. If yes to question 12, what were the observed responses to the non-drug intervention and how did the person change over time? 5. If yes to question 12, who brought up the non-pharmacological intervention as a treatment option? (e.g., physician, nurse, you) 6. If yes to question 12, what were you told about the effectiveness of non-pharmacological interventions to treat aggression/agitation? 7. If yes to question 12, what are some of the barriers you faced when non-pharmacological interventions were included in the care plan?    1. If no to question 12, what are some of the potential barriers you foresee as an issue to including non-pharmacological interventions to the treatment plan? 8. What is your opinion/concerns on using non-pharmacological interventions such as music therapy to manage aggression/agitation instead of being prescribed a drug? 9. How did healthcare practitioners monitor the progression of the resident’s agitation and/or aggression symptoms after administering the non-pharmacological intervention? 10. What drug approaches, if any, were discussed with you (caregiver or the resident) to manage agitation and/or aggression symptoms? 11. If approaches were discussed, what education or information did you receive regarding drug approaches for agitation and/or aggression? 12. Was there further testing needed to monitor the medications? 13. What were the observed responses to the medication and how did the person change over time? 14. How did healthcare practitioners monitor the progression of the resident’s agitation and/or aggression symptoms after administering the pharmacological intervention? 15. What reasons were provided, if any, to using drug approaches over non-drug approaches? 16. Did you have any concerns with using drug intervention used to manage aggression/agitation?     - 1. Are you aware of the advantages and disadvantages of this drug intervention?       2. What challenges do you perceive are associated with the drug intervention assigned to you/your resident? 17. How confident are you in the care plan developed by healthcare practitioners to address the resident’s agitation and/or aggression? 18. Is there something else that we have not yet discussed today that you feel is important to consider when it comes to the usage of non-pharmacological interventions to manage aggression/agitation? | - - 1. Goals, Intentions, Knowledge, Skills, Behavioural regulation     2. Reinforcement, Goals, Intentions, Memory attention and decision processes, behavioural regulation   2a. Emotions, Optimism, Beliefs about consequences, goals, knowledge, beliefs about capabilities, social influences   - - 1. Knowledge, Social influences     2. Beliefs about consequences, knowledge, social influences, optimism     3. Social influences, knowledge, social/professional role and identity     4. Knowledge, environmental context and resources     5. Knowledge, environmental context and resources   8a. Reinforcement, knowledge, skills  8b. Knowledge, skills, social/professional role and identity  8c. Optimism, beliefs about capabilities, beliefs about consequences  8d. Reinforcement, Behavioural regulation  8e. Social/professional role and identity  8f. Social/professional role and identity, social influences  8g. Optimism, beliefs about capabilities   1. Optimism, beliefs about capabilities 2. Reinforcement, Skills, Social/professional role and identity 3. Goals, Memory attention and decision processes   11a. Social influences, knowledge, environmental context and resources  11b. Reinforcement, goals, memory attention and decision processes  11c. Behavioural regulation, reinforcement  11d. Reinforcement, Skills  11e. Intentions, Goals, Knowledge, Skills, Beliefs about consequences, beliefs about capabilities  11f. Optimism, knowledge, beliefs about consequences, beliefs about capabilities   1. Optimism |

Thank you Statement: So, that brings us to the end of this interview. I’d like to thank you for taking the time to talk to us today and providing your insight on this topic. If you have any further questions or comments, please don’t hesitate to contact me or one of the other researchers. Thank you.

**Demographics-Related Questions (Asked to both healthcare practitioners and residents/ friends and family caregivers):**

1. Which of the following options best describes your **sex**? Sex is defined by the Canadian Institutes of Health Research (CIHR) as “a set of biological attributes in humans and animals. It is primarily associated with physical and physiological features including chromosomes, gene expression, hormone levels and function, and reproductive/sexual anatomy.”
2. Male
3. Female
4. Prefer not to say
5. Which of the following options best describes your **gender**? Gender is defined by CIHR as “the socially constructed roles, behaviours, expressions and identities of girls, women, boys, men, and gender diverse people. It influences how people perceive themselves and each other, how they act and interact, and the distribution of power and resources in society.”
6. Woman
7. Man
8. Non-binary
9. Gender fluid
10. Two-spirit
11. Prefer not to disclose
12. Other: please specify _______
13. What is your age group?
14. 18-34
15. 35-49
16. 50-64
17. 65-84
18. 85+
19. Place of Birth (Country):
20. Canada
21. Other: please specify Zimbabwe
22. Language(s) spoken, choose all that apply:
23. English
24. French
25. Punjabi
26. Cantonese
27. Mandarin
28. Arabic
29. Tagalog
30. Spanish
31. German
32. Vietnamese
33. Tigrinya
34. Other – please specify: Shona
35. Which of the following best describes your race, racial identity, or how you might be racialized, please select all that apply:
36. Arab
37. Middle-Eastern
38. Black
39. Chinese
40. Filipino
41. Indigenous
42. Japanese
43. Korean
44. Latin American
45. White
46. South Asian (e.g. East Indian, Pakistani, Sri Lankan, etc.)
47. Southeast Asian (e.g. Vietnamese, Cambodian, Laotian, Thai, etc.)
48. West Asian (e.g. Iranian, Afghan, etc.)
49. Mixed Race
50. Prefer not to disclose
51. Other – please specify _______
52. What is your role within long-term care, please select all that apply:
53. Resident or Patient
54. Family Caregiver
55. Family Physician
56. Nurse practitioner
57. Geriatrician
58. Psychiatrist
59. Nurse (RN, LPN)
60. Personal Care Aide
61. Health Care Aide
62. Manager
63. Other: please specify: Recreational Therapist
64. Approximate number of years in your role (e.g. resident, caregiver, provider):
65. 0-5
66. 6-10
67. 11-15
68. 16+
