# Supplementary material for: Barriers and facilitators to care for agitation and/or aggression among persons living with dementia in long-term care
Source: BMC Geriatr. 2024 Apr 11;24:330. doi: 10.1186/s12877-024-04919-0 (PMC11008022; doi:10.1186/s12877-024-04919-0)
Supplement: Supplementary file 3 — Supplementary Material 3. [file 12877_2024_4919_MOESM3_ESM.docx]

Additional File 3

Additional File 3.1: Barriers to Care at Detection and Diagnosis of Agitation and/or Aggression Among PLWD in LTC

| **Theme** | **Code** | **Quote** | **TDF Domain(s)** |
| --- | --- | --- | --- |
| Barriers in Administering  Diagnostic tests | Administering diagnostic tests may prove difficult because they are not adapted for persons with cognitive impairment | He was at the stage where testing was not possible anymore because he didn't understand the question to answer [it] (Participant 1) | Memory, Attention and Decision  Processes |
| Barriers in Administering  Diagnostic tests | Healthcare provider familiarity with agitation diagnostic tools affects comfort or competence with using tools | There are some tools that are used by mostly our nursing staff [...], those aren't things that I do assessments for so... and they kind of combine some of the mental health and behavior. So like, the DSM-T is one for example, that covers some of that. So, but none that I use as actual scales or tools. (Participant 8)  I think that the tool is probably only as good as the person using it. (Participant 10) | Skills, Knowledge, Social/Professional  Role and Identity |
| Barriers in Administering  Diagnostic tests | Difficulties among healthcare providers in understanding how agitation diagnostic tests works | Well, disadvantages, I actually don't know exactly what that scale is, or necessarily how they come with the number that they pick. So it's kind of a bit meaningless to me. (Participant 7) | Skills, Knowledge |
| Barriers in Administering Diagnostic Tests | Drawbacks of using tools to diagnose agitation (e.g. time consuming, healthcare provider availability, oversimplifying behaviours) | I think that the tool is probably only as good as the person using it. So it really is the responsibility of the staff member of the person using the tool in the moment to be able to accurately itemize or depict what is going on with that person because the person with dementia is agitated because they can't communicate their needs a lot of times. (Participant 10)  And I think oftentimes, there's not enough staff availability [...] so it's not even [just] getting the assessment done, [it] is [also] implementing the interventions that are identified [and] because we don't have enough staff, it can be a bit tricky. (Participant 9)  I think the disadvantage is […] every patient is different. And the tools don't really help you understand specific triggers or specific patterns of behaviors […]. It just literally tells you at this time of day, they were yelling, kicking, screaming, whatever. It doesn't necessarily help tease out what was going on around them and […] environmental triggers and that kind of stuff. (Participant 17) | Skills, Social/Professional Role and Identity, Environmental Context and Resources |
| Barriers in Administering Diagnostic Tests | Drawbacks to using tools to monitor agitation symptoms (e.g. not informative enough) | And like a lot of assessment tools, sometimes it becomes just a tick box, people are actually conscientiously thinking about their day, but not really paying attention to why it's being done and the change from baseline so it can just become a tick box. So those are kind of some of the disadvantages with nice tools. (Participant 3)  I feel like our assessment measures don't really tell us what type of intervention to use, more just tells us what's happening for the behavior, not like the cause, or what's going to help it. So I don't really think it's helpful for that in any way. (Participant 7) | Reinforcement, Beliefs about  consequences, beliefs about capabilities |
| Barriers in Administering Diagnostic Tests | Diagnosis of cognitive issues takes a long time, which delays diagnosis of agitation and/or aggression | So the whole process of diagnosis took about three years, and the cognitive neurologist was seeing us every six months, and she would test him every time with different mental tests (Participant 1) | Environmental Context and Resources |
| Barriers in Administering Diagnostic Tests | Lack of available diagnostic tests for agitation and/or aggression | And then when he was in [redacted] for a while, they did their assessment, and they [...] did sort of all kinds of assessments for him. [...] I don't think there was one specifically for agitation and aggression. (Participant 4)  Well, that we really only use one system, which is the BSMT. And I'm actually not really familiar with any other tools that we would be using. (Participant 11) | Environmental Context and Resources, knowledge |
| Challenges to diagnosis that relate to the person with lived experience | Diagnostic practices overlook hypoactive behaviours of dementia | The hyperactive usually attracts the attention of everybody because they're distressed, yelling, screaming, fidgeting, wandering, moving, so they're active, whereas the hypoactive, that's where people can be missed because they become quiet, sedated, in their bed not moving, and [are] more easily overlooked. (Participant 3)  So the diagnosis of agitation, aggression, they actually have to be more hyperactive. They have to have physical behaviors and signs so such as yelling and screaming, and wandering [...] and that they'd come to potentially harm to themselves or others. So it has to be enough that it's disruptive (Participant 3) | Skills, Knowledge, Emotion,  Behavioural Regulation |
| Challenges to diagnosis that relate to the person with lived experience | Residents' lack of awareness or expression | So he didn't believe it, because in his mind, it didn't happen. [...] He denied the whole thing and was very calm. (Family Caregiver/Recreational Therapist, Participant 4)  He's a "man's man". So he's always had that hard Macho Man kind of crust and he doesn't express how he feels about anything (Family Caregiver, Participant 2) | Emotion |
| Barriers related to diagnostic practices for agitation and/or aggression | Unclear awareness or availability of geriatric medicine or geriatric psychiatry services | Geriatric medicine I don't know actually, I'm not aware if they will come to a facility. I don't think so. [...] I certainly don't have any patients that are being actively followed by geriatrics on site. I have a couple patients that see geriatrics out in their offices, but I don't believe they come do on-site consult consultation. (Participant 17) | Social/professional role and identity, reinforcement, environmental context and resources, social influences |
| Challenges to diagnosis that relate to the person with lived experience | Residents are unique and agitation is especially unpredictable and fluctuates over time | “Sometimes that agitation and aggression can just come out of nowhere. So it's hard to really find a pattern sometimes. Like, there's always going to be that first time that somebody gets agitated or anxious. And it's hard to predict that.” (Participant 18) | Knowledge, skills, behavioural regulation, emotion |
| Barriers related to diagnostic practices for agitation and/or aggression | No formal criterion for agitation are used | I don't know if this really is a diagnosis. I mean, it's a behavior. So usually it says, "moderate dementia with this and this unkind behavior" or "see here dementia with this kind of behavior". And then going into detail, what kind of behaviors this person is showing, but it does not show up really as a diagnosis. So, it is always in combination with something. In this case, it will be in combination with dementia. (Participant 12) | Knowledge, skills |

Additional File 3.2: Facilitators to Care at Detection and Diagnosis of Agitation and/or Aggression Among PLWD in LTC

| **Theme** | **Code** | **Quote** | **TDF Domain(s)** |
| --- | --- | --- | --- |
| Process Facilitators to Diagnosing Agitation | Easy to administer agitation tools across different healthcare providers and produce easy-to-understand results | Well, the advantage is, it actually outlines the signs and symptoms, specifically for individuals, so that it's readily available and reproducible. So you know, and somebody who's unskilled can actually use a lot of these tools. So that's the good thing (Participant 3)  One advantage to the BSMT is it kind of guides you towards what interventions have worked and what hasn't. And what kind of behaviours you're having and whether a treatment like a medication will help or if you need some other type of intervention. So it'll tell you which ones may respond to antipsychotics and which ones won't. (Participant 15) | Optimism, Beliefs about capabilities |
| Care team facilitators to detection and diagnosis | Specialized care teams helped with diagnosis | I have their dementia care team that came and interviewed and I had respite care for a while. And then there was a community service person who came to see me. And yeah, so that was the diagnose to get him into care. (Participant 1) | Social/Professional Role and Identity, reinforcement |
| Process Facilitators to Diagnosing Agitation | Use of tools for diagnosing agitation and/or aggression symptoms (e.g. BSM-Ts (daily behavioural mapping tools), RAI assessment etc.) | So, for some of the behaviors depending on if a person is kind of experiencing regular periods of increased agitation, protective behaviors, then they'll do they'll use BSM-Ts, I guess I probably should have said BSM-Ts too, as primarily nursing and would do that nursing physicians to track and monitor over a time period. (Participant 10)  Ah, yeah, I mean, right now, it's sort of daily behavioral mapping (Participant 3)  We use the RAI. And I believe there's one specifically on the RAI related to that, I can't remember what it's called. So we get that number, our RAI assessor figures out that number for us, and reports it when we renew that person every quarter. We try and keep that to a certain number and not, like, too high. (Participant 7) | Goals, Intentions, knowledge |
| Process Facilitators to Diagnosing Agitation | Advantages to using a diagnostic test (e.g. being able to compare agitation between residents, objective measures) | Advantages [are] that [...] you can kind of pick a number to compare between different residents. And once it gets to a certain point, you know you really need to put in some intervention there to try and like lower it ( Participant 7)  I really enjoy exploring the non-pharmacological approaches. I do think that the sheet that we uses asks quite a bit of questions [...] so I do think it is helpful. (Participant 11)  I think the advantages are that [...] it keeps the assessments somewhat objective when you're just commenting on exactly what behaviour you're seeing (Participant 17) | Beliefs about capabilities |
| Process Facilitators to Diagnosing Agitation | Preference among healthcare providers for screening tools | In long term care, somebody can present in many different ways. So that's why I like those general screening tools. (Participant 3)  So trying to have people educated with as many tools as they can, so that they can identify agitation, the non-verbals, so to limit escalation, hopefully, of those behaviours. There's different scales, these primarily nursing, [...] will give us an indication as to agitation for people, when people will become more agitated. Suicide assessments, pain assessments [...] (Participant 10) | Beliefs about capabilities |
| Process Facilitators to Diagnosing Agitation | The high volume of assessments for other behavioural issues is part of the assessment for agitation | So trying to have people educated with as many tools as they can, so that they can identify agitation, the non-verbals, so to limit escalation, hopefully, of those behaviours. There's different scales, these primarily nursing, [...] will give us an indication as to agitation for people, when people will become more agitated. Suicide assessments, pain assessments [...] (Participant 10 | Reinforcement |
| Care team facilitators to detection and diagnosis | Providing adequate training for healthcare providers to use agitation screening tools | So trying to have people educated with as many tools as they can, so that they can identify agitation, the nonverbals, so to limit escalation, hopefully, of those behaviors. (Participant 10)  And then it can be fired up to train staff or train staff, who if they need to, can have the [...] screening tools. (Participant 3) | Skills, Knowledge,  social/professional role and identity |
| Process Facilitators to Diagnosing Agitation | Counting number of aggressive or agitated incidents to diagnose agitation | Looking at incidents of aggression in the person, if they're consistently happening like every day or every week, then [...] you would think that there's something that we need to make note of, and put on their file, which would also relate to the number that the RAI assessor gives for the aggression. And I think when it hits a certain point, then that has to go on their care plan as an issue. (Participant 7)  Typically, what we look at is [...] instances of documented experiences within electronic care plans. And so getting to kind of compile those over, you know, whatever month or review period, usually combined with our RAI assessment [...] (Participant 8) | Skills, intentions, goals |
| Process Facilitators to Diagnosing Agitation | Involving geriatric medicine or geriatric psychiatry (e.g. on a referral basis) | When the diagnosis is becoming vague. And so if I'm not absolutely sure what's going on, and I want a second opinion, [then it's] very helpful to have them involved, because it's a colleague who can assess and just bring in a different perspective. (Participant 3)  Only on a referral basis. So if there's like someone that needs like specific consultation because we can't figure out how to manage it, then we do. (Participant 7) | Skills, knowledge, social/professional role and identity |
| Process Facilitators to Diagnosing Agitation | Less referrals needed in LTC centres where physicians are more actively involved in care | Some places have physicians who are more actively involved in care, like, present more often and more actively involved. And then sometimes that seems to reduce aspects of some of those needs, because they're, especially in terms of med management, able to be more present to respond more quickly. (Participant 8) | Environmental context and resources,  social/professional role and identity |
| Process Facilitators to Diagnosing Agitation | Diagnosis is made by a physician | So the diagnosis comes from the doctor, ultimately, but the doctor gathers that information from the staff charting and […] observed behaviors. (Participant 9)  Usually [the] physician would be the one to diagnose it. (Participant 15) | Social/professional role and identity |
| Process Facilitators to Diagnosing Agitation | Diagnosis for agitation and/or  aggression is during the process of the dementia diagnosis | it's usually related to a diagnosis of some sort, such as Alzheimer's type dementia, or something of that sort. So once we have a descriptive scenario, we would use that as a describer itself [of agitation]. That's how we get there. (Participant 16)  Making sure that we're ruling out any other causes. So we're doing blood work if it's safe to do so. Reviewing their medications, [...] files, [...] [and if] there's any triggers. Making sure there's no infections or so other organic causes. And then you can say, 'Okay, well, this is likely from dementia', or like a mix of dementia and depression, or dementia and agitation, anxiety. And then we can go down - 'Okay, well, then, how should we be treating this?' (Participant 18) | Reinforcement, Knowledge, Skills |
| Process Facilitators to Diagnosing Agitation | Reviewing experiences of all care team members working with the resident to create a diagnosis of agitation and/or aggression | Well, we have staff working with these people, you know, throughout the day, so their experience is a huge factor. So reviewing the notes, and what people are saying. (Participant 16)  Nurses do it. I go in there once a week and I just [...] ask about orientation, sir name, 'Where are you?'. Yeah, but [...] I [mostly] just go by "Hey, how were they responding to me before? And how are they responding to me now?". (Participant 18) | Knowledge, Social/Professional  Role and Identity, Reinforcement |
| Process Facilitators to Diagnosing Agitation | Documenting and recording agitation events in many places | “just like nursing charts, healthcare aid charts, in their hand over in their communication to me, behavior mapping.” (Participant 18) | Knowledge, skills, reinforcement, goals |

Additional File 3.3: Barriers to Care at Care Coordination and Management of Agitation and/or Aggression Among PLWD in LTC

| **Theme** | **Code** | **Quote** | **TDF Domain(s)** |
| --- | --- | --- | --- |
| Barriers involving care team members | Lack of action among care workers | And I truly believe the care manager didn't care. She would say one thing, but it never, never happened (Participant 1)  They were like, "they knew everything" and it was their way and that was the way it was, you know. Relatives, friends, nobody had any say [...] and they were going to be treated the way they were treated, period. (Participant 5) | Social/professional role and identity,  Beliefs about capabilities |
| Barriers related to care  practices | Constantly changing directives in LTC facilities for agitation and/or aggression | And I mean, as per the guidelines, they changed over the years, I've had to change, what's my first line of what I will actually pick. (Participant 3)  But I have to say that our business, it never fails to surprise me six weeks, six months later, something new has come up. (Participant 3) | Environmental context and resources, knowledge |
| Barriers related to care  practices | Lack of directives in LTC facilities for agitation or aggression | Like, I'm not feeling like they really have a system for that or like, I don't know, I would expect to see, you know, a checklist of different things you could try, right? (Participant 2) | Environmental context and resources, knowledge |
| Barriers due to resource constraints | Cost of care barriers | And then it depends as well on costing, I mean, fortunately, most of these are now covered, but they weren't always. So it comes on "what's Blue Cross cover[ing] now"? (Participant 3)  Money, of course, [...] will always be the issue, because [more money is needed for] more interventions, but the other piece is the ability to administer the intervention. (Participant 9) | Environmental context and resources |
| Barriers related to care  practices | Cultural and language barriers to care for agitation among POC residents | I think cultural. I think everyone did realize that there would be some cultural barriers. [...] [T]he system is not really made for other people other than the whites or the majority population in the country. So you have to navigate the system with that lens, and understanding that you need to be proactive in saying what your cultural preferences [are] and what their meanings might be for the person that they're caring for. (Participant 6) | Environmental context and resources |
| Barriers related to the resident's emotional or physical needs | Decline in mental status or  increased dementia impeding care for agitation | That weekly meaning is excellent. We sit down with the physician, and [...] he's on medication for his memory, which is great. And sometimes they wonder if they should take it off. And I'm always like, no, please don't, because we'll probably see a rapid decline, and you can never regain. (Participant 4)  So I've had a few people that way it's, you know, it's not their dementia, it's [their] underlying paranoia. Yeah. And it's been a long standing, but the dementia can sometimes just destabilize them enough that, you know, they may have compensated for 10 years or whatever without [them but] now they're actually needing pharmacological management to help them cope. (Participant 3) | Emotion, Memory attention and decision processes |
| Barriers related to the environment | Environmental challenges (e.g. loud noises, set-up) | Loud noises - of course, being in a care facility that is specific to dementia, there's a lot of people who make noises, they scream. They, you know, they can push you because they don't understand that [it] can bother you. (Participant 1)  And so I think it's interesting, too, that other dementia units have their doors or, you know, have nice murals on them to look like they're part of the building and not a door. And so that's something they don't have. (Participant 4) | Environmental context and resources |
| Barriers related to family and friend caregiver preferences and values | Caregivers may not always understand how agitation and/or aggression impacts patient care | [Family member] may not understand what my intervention was. And so like, she wants him to have a shower. She wants him to have his beard shaved. She wants all these things. (Participant 4) | Intentions, Goals, Social/professional role and identity |
| Barriers related to family and friend caregiver preferences and values | Caregivers may under-report symptoms | And she wasn't telling us [about the resident's behaviour] because she was trying to protect him. (Participant 4) | Intentions, Goals, Social/professional role and identity |
| Barriers involving care team members | Lack of communication (staff-to-staff, staff-to-caregivers) | Well I guess they have talked to me about [...] his reluctance to participate in activities and showers and things like that, and how [...] they do step back and try again the next day or later on in the day. But that's about it. So my frustration is the lack of communication between the nurses and healthcare aides and the next shift that comes on. (Participant 2)  I think the communication is not the greatest. Especially when it comes to the agitation, I usually have to go to them and say, you know, how come he's in his clothes for four days? And then they'll say, Oh, well, he's not letting us change, or he's hiding his clothes and put on the dirty ones before we get into the room kinds of things like, they don't call me up and say, "Hey, we're having problems". (Participant 2)  I think I mean, honestly, it [is] primarily initially just nursing assessments and rapport, and then behavioral mapping, per sort of the usual protocols, depending on the facility would be the big one. (Participant 17) | Social/professional role and identity, knowledge, environmental context and resources |
| Barriers involving care team members | Lack of communication between health facilities | So the previous facility might not even send us any information, on what works and doesn't work, like a quiet place, or dim lighting, or what's the best way to redirect them, so whenever that inter-facility transfer happens, we're restarting all over. (Participant 11) | social/professional role and identity, environmental context and resources, Goals |
| Barriers involving care team members | Lack of competency among staff to deliver care | I don't have a lot of confidence sometimes in social workers. And I'm not sure where she was coming from other than they were probably at overcapacity (Participant 4) | Skills, knowledge, social/professional role and identity, beliefs about capabilities |
| Barriers involving care team members | Lack of coordination of care among team members in LTC | So my frustration is the lack of communication between the nurses and healthcare aides and the next shift that comes on. That's where they're not aware that 'Oh, he didn't, you know, change his clothes for four days' sometimes. (Participant 2) | Skills, knowledge, social/professional role and identity, beliefs about capabilities, reinforcement |
| Barriers related to family involvement | Lack of education among friend and/or family caregivers for caring for agitation among PLWD | No [...] it was just the initial application form, or orientation forms that I filled out when [resident] started there. And [...] sometimes additional measures are required in order to assist the residents in being comfortable. And these are some of the measures that we offer and it was just check a box if you're accepted, with once you accept, right? And that was with a social worker, it wasn't even with a nurse or anything. (Participant 2) | Social/professional role and identity, knowledge, skills, environmental context and resources |
| Barriers related to family involvement | Staff only have personal background knowledge as their training, with no extra education from long-term care | Most of the education I received, I received through my studies and working with people, but really not much from the home other than in the wellness meetings. And they're only yearly, so. (Participant 5)  The rest of [my education] has come from my own personal experiences and training I've had outside of being a nurse. It's actually been in the community through [...] FSCD - families with disability services. (Participant 11) | Beliefs about capabilities,  social/professional role and identity, skills |
| Barriers involving care team members | Healthcare practitioners are not raising awareness of issues for fear of job (e.g. termination, etc.) | I think it's really hard for staff to go into those parts of it, like, employers don't like it. Yeah. And I wouldn't want them to put... because I worked in long term care, I wouldn't ask those questions, because I wouldn't want them to put their jobs at jeopardy. (Participant 5) | social/professional role and identity, environmental context and resources, social influences |
| Barriers involving care team members | High staff turnover in LTC | And there seems to be an awful lot of new people or relief staff on and [...] unfortunately, we're in a time of COVID. (Participant 4) | environmental context and resources, social influences |
| Barriers related to family and friend caregiver preferences and values | Lack of inclusion of needs and values of family and residents | They were...like "they knew everything" and it was their way or and that was the way it was, you know, relatives friends, nobody had any say but these were little prisoners in their little prison, and they were going to be treated the way they were treated, period. So that... it was horrible actually. (Participant 5) | social/professional role and identity, beliefs about capabilities, beliefs about consequences |
| Barriers related to care  practices | There is a lack of personalized care plans and thus low confidence in care | It's kind of a hit or miss thing, whether they actually will be ready at that specific time to shower [the resident]. So we've stopped doing that, but, you know, we're willing to work with them, but we just can't seem to be consistent with anything. (Participant 2)  I don't even know that [a care plan] exists. So I would say my confidence level is pretty low. (Participant 2) | social influences, environmental  context and resources |
| Barriers related to care  practices | Lack of attention to hypoactive behaviours | The hypoactive, that's where people can be missed. Because they become quiet, sedated, in their bed not moving, and is more easily overlooked (Participant 3)  So these things aren't caught? Because "he's not a problem at the moment". [...] And he doesn't act out physically, he's not throwing things [...]. So I just think that, you know, the squeaky wheel always gets the grease kind of thing. And he's not the squeaky wheel, right? (Participant 2) | Skills, knowledge, intentions,  social influences, behavioural regulation |
| Barriers related to care  practices | Lack of follow-up of patient agitation symptoms | I think they deal with it okay, but it's just the lack of follow through and follow up to deal with it again, the next time. (Participant 2) | Reinforcement, Intentions, Goals |
| Barriers involving care team members | Lack of training for health care providers caring for agitation or aggression among PLWD | I think the concern for me would be staff not having enough education around the terms agitation or aggression. And then how do you manage that? [...] And so a lot of it is around the education piece, and that's what I find when I'm on that unit. Specifically, that they don't seem to have any skill set. (Participant 4)  [A] lot of staff go into a nursing home and have no idea how to deal with aggressive people and tend to become aggressive back, and that just multiplies the situation, so I think education of staff too is a real key. (Participant 5) | Skills, knowledge, beliefs about capabilities |
| Barriers involving care team members | Inconsistent training for health care providers caring for PLWD with agitation | And there seems to be an awful lot of new people or relief staff on and again, you know, unfortunately, we're in a time of COVID. But I have no idea at that building, if they get any services or education, or they, you know, and I guess a lot of them seem to me to be very new at what they're doing. And you have to learn that way. But is there somebody who's mentoring them? (Participant 4) | Skills, knowledge, beliefs about  capabilities, social influences, environmental context and resources |
| Barriers due to resource constraints | Not enough time for health care providers to provide care | There isn't enough time, and enough staffing and enough time in nursing homes to give the kind of person centered care that would really remedy the situation. (Participant 5)  And I mean, health care aides are most plentiful, will do as much as they can. But they've got limitations in terms of their availability and time. And so it's sort of that triaging for 'who's most in need at this moment?', which maybe doesn't help in the long run. (Participant 8) | environmental context and resources, social influences |
| Barriers related to care  practices | Difficulties among LTC staff to understand the residents’ needs (e.g likes, dislikes) | Sometimes it's a difficulty of, again, trying to figure out the extent to which like, is this some sort of environmental need, is the person agitated for reasons that we can actually attend to? And then trying to distinguish when you're trying to work with whatever type of intervention and so in situations like that, can be challenging. (Participant 8)  the 'it not working' can also be because there's no background on an individual, there's a long history of disconnect, so trying to learn more about what would be important to somebody can be really hard. (Participant 8) | Behavioural regulation, Emotion |
| Barriers related to care  practices | Reliance on caregiver as management strategy for agitation | we're still massively relying on caregiver to manage the disease. (Participant 3) | Environmental context and  resources, social/professional role and identity |
| Barriers related to the resident's emotional or physical needs | Resident personal qualities as barrier to care | I think a lot of times that if you decide that, you know, the medication would help this person's needs to have more activity or have more routine or have more structure or have more input from the team day to night then...sometimes if they're, if their history in the community is very isolated, very self-isolating, and they don't have that history of participation, or joining or even communication. Trying to get that now, during a period of agitation, is really challenging. (Participant 10) | Emotion, Memory attention and  decision processes, behavioural regulation |
| Barriers related to the resident's emotional or physical needs | Residents having difficulty communicating needs | We get a lot of people who will have increased agitation because they're highly anxious, and they're afraid of being alone. And so they're not able to communicate that need, but it's being [...] an investigating detective to figure out what is the trigger. (Participant 9) | Memory attention and decision  processes, emotion |
| Barriers due to resource constraints | Lack of available resources | And it depends what resources are available. So you have to look at the person, the site, their history, what may or may not work with them, and what's simply available within the system. So, yeah, they don't often have a choice, sometimes it's what and whoever is available. (Participant 3)  We don't have enough staff. And [we] don't have enough resources. [...] We can't create as much of a home environment [...]. (Participant 18)  So to have the staffing, to have the resources and then time - that's the other piece. We're only hired for so many hours in a day, and you can only have so many staff, but it's having that cohesiveness of staffing to have the time to provide the intervention when the client needs it. (Participant 9) | Environmental context and  resources |
| Barriers involving care team members | Healthcare providers are not communicating care practices with residents | I have to tell aides, you know, 'Back off, tell her what you're doing. Because this is... she's protecting herself. She's not trying to attack you for any other reason than, she's protecting herself'. (Participant 5) | Behavioural regulation, intentions, goals |
| Staff Characteristics as Barrier to Care | Certain staff members unable to carry out care due to personal characteristics | In the care facility, what would really agitate him was male caregivers (Participant 1)  sometimes you actually have to change people because, in fact, they're a trigger for their agitation, or whatever reason. (Participant 3) | Social/professional role and  identity, social influences |
| Barriers due to resource constraints | Staffing issues | So there was one LPN, and three healthcare aides for 30 patients with dementia. It wasn't enough. (Participant 1)  And then another barrier would be staffing. Staffing is a big one, so on my unit, I'd be the only nurse for about 23 to 26 [residents] [...] and then there's three healthcare aides. And we are very often short. (Participant 11)  Having the adequate staffing ratios, being in an environment that is focused on dealing with that kind of condition (Participant 6) | Environmental context and  resources |
| Barriers related to care  practices | Too many staff members handling a patient resulting in agitation | Some of the nurses, myself and a couple other aides [found it] okay to have the four [staff members] there. But it was too many [that] it increased agitation with [the resident]. So if you had two [staff] kind of keep out of sight, in case the aggression was more than the caregivers could handle, [then] the less people [there made the resident] much calmer, much less aggressive. (Participant 5) | Environmental context and  resources, beliefs about consequences, skills, social/professional role and identity |
| Barriers related to the resident's emotional or physical needs | It is important to look for triggers, contributing causes, and unmet needs that lead to agitation and/or aggression | So they can tell when it's really painful, and that will increase his agitation and aggression. He's got pain because he can't communicate to them that it hurts. (Participant 1)  Sometimes it's a difficulty of, again, trying to figure out the extent to which, is this some sort of environmental need, is the person agitated for reasons that we can actually attend to? And then trying to distinguish when [to use] whatever type of intervention and so in situations like that, can be challenging. (Participant 8)  Making sure that we're ruling out any other causes. So we're doing blood work if it's safe to do so. Reviewing their medications, reviewing their files, reviewing if there's any triggers. Making sure there's no infections or so other organic causes. And then you can say, 'Okay, well, this is likely from dementia', or like a mix of dementia and depression, or dementia and agitation, anxiety. And then we can go down - "Okay, well, then, how should we be treating this?" (Participant 18) | Memory attention and decision  processes, emotion |
| Barriers related to care  practices | We can identify an unmet need, but there can be difficulty with solving it | Sometimes you can identify the need, but then solving the need is also a challenge because the person's already agitated. So you know, you determine what that person needs. The road to the solution is because they're agitated, you can't get there either. So finding a new path to the solution. (Participant 10) | Goals, intentions, environmental  context and resources |
| Barriers involving care team members | Not all LTC sites have access to necessary interdisciplinary team members | I even hear nurses at my work, sometimes they're like, "We need more nurses. So we need to like cut the recreation program". And I'm like, okay, I can understand that you think there's a lot of people that work in the rec program, and yeah, we do need another nurse. But I feel like then they're not quite understanding the important role that recreation has in the enrichment. (Participant 11) | Social/professional role and identity, Beliefs about capabilities |
| Barriers involving care team members | Staff from different cultural  backgrounds respond differently to agitation | And then often what I see as well is, like, we're working with large diversity of staff from different cultural backgrounds. And so, what I see as well is that their response is very different towards behaviours. (Participant 12) | Social/Professional Role and identity, knowledge, skills, social influences |
| Barriers involving care team members | Different healthcare providers perceive planning of care to be specific to their professional roles | Well, we don't administer that. That's nursing and nursing is an independent profession. So they make nursing plans, because they're facing the same issue that we are. So we do our part, they do their part, we don't tell them what to do. And quite often, we try to coordinate measures, even if you start a pharmacologic approach, you still need to have a staff approach. That needs to be supervised and reassessed from a nursing point of view. And, you know, they have the many tools, you know, the tests and the rugs, and all these other tools that nursing uses to keep track of things. (Participant 16) | Social/Professional Role and Identity, Knowledge, Skills |

Additional File 3.4: Facilitators to Care at Care Coordination and Management of Agitation and/or Aggression Among PLWD in LTC

| **Theme** | **Code** | **Quote** | **TDF Domain(s)** |
| --- | --- | --- | --- |
| Facilitators related to care  practices | Appropriate Use of Antipsychotics as helpful for pharmacological use guidelines | I think if anything, quite a few years ago when they started [...] the AUA - the appropriate use of antipsychotics. That has been quite a helpful, I think, intervention in that it really makes people look at, and be accountable for, pharmacological uses that they have in the building. (Participant 10) | Behavioural regulation |
| Facilitators related to care  practices | Changing language around agitated and aggressive behaviours to be more patient-centred | One thing is to change our language too, so we try not to use aggressive or abusive in terminology. […] we're trying to follow that GPA model and really use that (Participant 10) | Goals, knowledge, intentions |
| Facilitators related to external supports | Hiring someone to carry out interventions or day to day affairs with resident | Can the family pay for somebody to come in? And if they can, well, then then they'll hire somebody who will come in, walk with that person for three hours every day, and take them out and do things and then that resident really gets to know that person. (Participant 8) | Social influences, goals, intentions, environmental context and resources |
| Facilitators related to care  practices | Healthcare providers need to be able to self-regulate when providing care to aggressive residents | I think the only things that I would be unhappy with are like, a lot of physical intervention, you know. I don't want somebody being aggressive in return. Does that make sense? Aggressively trying to stop aggression? Yeah. (Participant 5) | Skills, knowledge, beliefs about  consequences |
| Facilitators related to  environmental factors | Environmental benefits of the facility (e.g. supportive environment, personalized environment) | At [LTC centre], they have an atrium floor and [...] relaxing places where people can go [...] where even an aide or a nurse can take someone to get them away from the other people and the noise and bustle, because a lot of noise can be irritating too! [...] Sometimes it's nice to be able to get away. So quiet places, quiet music, that type of thing. I think that, and having those spots, really makes a difference. (Participant 5)  I guess external, depending on how far you're measuring that, [...] might even be just like what's available in a building or what the building is designed like (Participant 8) | Environmental context and  resources |
| Facilitators related to family  involvement in care plans | Using family members to help provide care | Family members - they will give us obviously the biggest part of history. And then they will also give us just ideas of changes in behavior. (Participant 10)  And then it's getting family up in there. At times [...] we're calling the family we're talking to them on the phone, or video, or they're coming on site. Because that's the only way we're gonna get [the resident] settled in an acute situation without having to chemically restrain them [...]. So family or people they know, or friends can be really critical. (Participant 3) | Social/professional role and identity, beliefs about capabilities  Intentions, goals |
| Facilitators among care team members | Communication with  staff is effective among persons involved with the care or planning of care in LTC | So that was very well explained both by [...] our family doctor, the cognitive neurologist, both care facilities, the DOP nurse like, they were very good about explaining that - what it meant. [...] I think that they were really good about following up and taking my input (Participant 1)  And then when [the resident] was in the hospital, they were really good with telling me everything that they were using. (Participant 1)  And then that gets communicated among the team, as well as incorporated into their care plan. [...] finding ways to communicate so that the team knows in a timely manner, how to provide that support, because when someone is triggered, you could be anywhere really. And we want to be able to provide those creative ideas without having to run back and read a computer term, for example. (Participant 9) | Social influences, social/professional role and identity, reinforcement |
| Facilitators among care team members | Adequate training is provided to increase competency and expertise among LTC staff | I'm very confident, you know, I feel very confident with the staff, and with the doctors in in this location, very much so. (Participant 1)  And I think the fact that the staff [are] educated well, in how to deal with those kinds of patients, makes it a really good place and makes me comfortable knowing that's where [the resident] is. (Participant 6) | Skills, knowledge, beliefs about  capabilities |
| Facilitators among care team members | Team members coordinate care between each other | So whatever is needed in that situation, everybody will go and assist when they're called upon or when the need arises. [...] the team dynamic is such that we understand that if I raise my hand, call HELP, it's not that I'm not skilled, or I don't have the ability, it's that I need help. [...] Or maybe I'm not the best person for the situation. ( Participant 10)  when I need to find out what exactly is going on, I need to get everybody who's right there in real time in, you know, in with the residents to try and actually figure out what may be triggering and how to manage. So yeah, I find it full involvement with the families, agents, health care aides to make sure that we're getting that full picture and their input. (Participant 3)  It's important for everybody who's involved, to know all the interventions that are being used. So it's important to know this person has antipsychotics in place or a history with mental health or high agitation or depression or suicidal ideation, it's important for all team members to know that. It's also important too for all team members to know that during periods of agitation, they like to go in the garden or like to watch western movies or those quick things that you can do to reduce the agitation. [...] Everybody should know both sides, pharmacological and non-pharmacological, no matter what side of that job description you're on (Participant 10)  In a sense, I'd say everybody, in some aspect or another, right from the healthcare aides for [...] helping the person get dressed or shower them or toilet them - may incorporate some of those aspects [...] whether it's playing music or trying to work within what the person is most comfortable with, or the people that are most comfortable with [...] Everybody, again, from their own discipline has the chance to help with that, or more work with the residents (Participant 8)  That is being assessed by, firstly, by a nurse, and then the nurse will communicate that with the doctor. But in the first place, we, as an HCA, we're the ones seeing it in the first place, first thing in the morning. So it's very important to communicate that to the nurse so that the nurse can communicate to the doctor - whatever assessment that specific person needs for assessment or reassessment maybe. (Participant 13) | Reinforcement, social/professional  role and identity, goals, intentions |
| Facilitators among care team members | Interdisciplinary or entire team used to develop care and management plans | Hopefully it's kind of every team member. So we do have our interdisciplinary team that regularly debates and we discuss each resident several times a year, and then more so if needs arise. And so it's anywhere from HCA to physio, TRT, social work, dietary, the entire interdisciplinary team. Family members (Participant 10)  All of them. All professional disciplines. I mean, right through social work, I mean, everybody: psychology psychologists, yeah, nurse practitioners, physicians. I find it can take every one of us. (Participant 3)  And it really involves family as well, family, friends, and even volunteers. We have volunteers and paid care aids that will actually come in as well to help support all that. So yeah, it's all hands on deck. (Participant 3) | Reinforcement, social/professional  role and identity, goals |
| Facilitators related to external supports | LTC have committees or groups that help to provide the best evidence to inform care | So we have working boards or working committees who provide information, right so we can make knowledgeable informed decisions and choices. (Participant 9) | Social/professional role and  identity |
| Facilitators related to family  involvement in care plans | Education of friend and family caregivers enables better agitation and/or aggression care among PLWD in LTC | As far as the medications are concerned, the pharmacist and the doctors would discuss with me before any of the medications were started. (Participant 6)  Gave us written information. And then we had a meeting where all the people I've mentioned before were in attendance, and then discuss the different interventions. Like it was really thorough and the information was provided at a level that a non-medical person would understand, which is great for [family member]. (Participant 6) | Skills, knowledge, beliefs about  capabilities, social/professional role and identity |
| Facilitators among care team members | Education among healthcare providers for management approaches for agitation and/or aggression enables better care | Where he is right now they're very well educated and continually are educating their staff on different ways (Participant 1)  And I think the fact that the staff [are] educated well, in how to deal with those kinds of patients, makes it a really good place and makes me comfortable knowing that's where [the resident] is. (Participant 6)  I think one of the resources is that training we got on how to deal with aggression. I think recently I got an invitation to a webinar on it. But other than that, I'm I haven't really come across much. (Participant 7)  Because we do have training that when it's escalating on how to do nonviolent crisis intervention. [...] so how to hold them or restrain them to be able to safely get them to a place where they can be settled and remain safe. So yeah, non-violent crisis intervention. So we were all trained to those techniques as well. (Participant 9) | Skills, knowledge, beliefs about  capabilities |
| Facilitators related to external supports | Hiring recreational therapist privately for residents with agitation | So a recreation therapist is very focused. So what are we going to do? What did [the resident] like to do? So we would provide her with all this information. And so then when she comes, she's very focused about, okay, this is what we're going to do. Let's go play cards. Let's go play crib. And [the resident] loves it. [They have] a great time. (Participant 4) | Social influences, goals, intentions, environmental context and resources |
| Facilitators related to family  involvement in care plans | Personalized and interdisciplinary approaches to care improve confidence in care plans | They listened to me, they explained what could happen, how it should work. So I had a lot of good information. (Participant 1)  It's very much a team effort. They have asked us for songs that we think might help [the resident] calm down. We've given them some of the words in our language to try and calm [them] down. (Participant 6)  In the location that [resident's] in now, I'm quite confident. The fact that they have [...] non-medicinal programs to deal with aggression, the fact that they have staff that seems to care about the individuals who are interested in person-oriented care. [...] and they try to find out about the individual there, their previous life, so they have an idea where the aggression may be coming from. (Participant 5)  I didn't want [the resident] being on food restrictions [...] if [the resident] needs a cookie, give [them] a cookie. Okay? If that makes [them] happy. (Participant 4)  [LTC centre] does an excellent job with I think their care is quite patient centered. (Participant 5) | Social/professional role and  identity, intentions, reinforcement |
| Facilitators related to deciding on care plan | Care plans for all interventions need to be tailored and patient-centred | I think if you know the resident and have the right information available from family about what is likely to work, and I think that most non pharmacological interventions are fairly low risk. (Participant 17)  I think that getting to know them, getting to know what their triggers are, trying to avoid those and understanding what kinds of things calm them. That might be a doll, it might be a blanket, it might be a snack, it might be a song, right (Participant 2) | Intentions, Goals, Behavioural Regulation, reinforcement |
| Facilitators related to deciding on care plan | The focus of the care plan needs to align with goals of care for the resident | So I think it's just [...] sometimes just reminding [staff] that we're not going for cure, because there's no cure for what [the resident] has. But we're going to keep this [individual] safe [...] and happy. (Participant 4)  I think after trying a few different [drug] combinations, we settled on one which is now kind of, not modifying really the behavior but in a way, controlling and tempering down the behaviors. (Participant 6) | Goals, optimism, intentions,  beliefs about capabilities |
| Considering precipitants for a  diagnosis of agitation | Having a checklist of precipitants to consider (e.g. basic needs: food, drink, pain, medication, etc.) aids in management plans | So that's kind of like the first go to certainly for agitation, if you notice that a person is just increasingly or suddenly becoming agitated you want to get to those basic needs first. Is it pain? Are you hungry? Do you have to go to the bathroom? What are those basic needs? Are they being met? (Participant 10)  if there's been a change in environment, or a loss. Yeah. So we're actually looking at precipitating factors that can contribute. Some of the subtle or subtle ones can be pain. So, and then hunger and then you know, there's physiological abnormalities and diagnoses that may be masking this agitation, particularly for somebody with dementia. (Participant 3) | Skills, Emotion, social influences,  environmental context and resources, behavioural regulation |
| Facilitators among care team members | Residents respond better to some staff members and disciplines than others (e.g. rec therapy) | Some [residents] respond better to particular individuals or disciplines than others, or to certain things, whether it's going for a walk or being outdoors or sitting with them, reading to them, or engaging in different music stuff. Getting to, as a team, collaborate and be able to identify those and then say, 'Oh, she has really good success with Rec or with that person in the rec team. So you take her out and go do that' (Participant 8) | Social influences, Emotion, Memory attention and decision processes, behavioural regulation |
| Facilitators related to care  practices | Ensuring that the follow-up of agitated symptoms is  integrated into care practices | They are supposed to document that. So that you get a good impression about, timelines, for example. When are the times where this person is most aggressive? Like the documentation of the acceptance of this person towards care. How many staff were needed for care, or is the person compliant, does the person have issues, and things like this. (Participant 12) | Reinforcement, Knowledge,  Intentions, Goals |

Additional File 3.5: Barriers to Care at Acute/Severe Treatment of Agitation and/or Aggression Among PLWD in LTC

| **Theme** | **Code** | **Quote** | **TDF Domain(s)** |
| --- | --- | --- | --- |
| Acute and severe agitation  treatment | Reliance on physical and/or chemical restraints | I think like in the severe cases, sometimes we have to physically hold the person or remove them, or sometimes give them an emergency pharmacological restraints at the time. I think in some cases too, like an emergency, a mechanical restraint may be used, they might be given a seat belt in a wheelchair, or something, if it's a really bad case. (Participant 7) | Behavioural regulation,  reinforcement |
| Barrier for non-pharmacological treatment for acute agitation | Agitation symptoms are too severe (e.g. safety concerns) | When a person is in that extreme agitation [...] you've determined that this is the immediate course of action [...] to get Haldol [or] Seroquel, whatever, into that person. (Participant 10)  Well, I have a huge fear that [the resident's] going to hurt someone, right? And 98% of the time, [they] can be redirected. But that other 2%, without the medications that [they're] on, they wouldn't be able to redirect. (Participant 1)  And sometimes [...] the need is present and urgent. And so we need something to work quickly because somebody else will get hurt if we don't act sooner. And so that can be another reason. (Participant 8)  "There might be a PRN med that can be administered" (Participant 8) | Skills Behavioural Regulation, Goals, Intentions, Beliefs about consequences |
| Acute and severe agitation  treatment | Takes time to acquire consent for a mechanical restraint | So another barrier would be when a resident is requiring a physical restraint, there needs to be a physician's order. And so we have on call physicians, we don't have one on site, but we do have on call, and then that can be a barrier as well, because that takes time. (Participant 11) | Environmental Context and  Resources, Social/Professional Role and Identity |
| Acute and severe agitation  treatment | Acute/severe agitation can warrant emergency services | I mean, long term care, it's quite limited. I would say very worst case scenario you call paramedics or police for help. (Participant 17) | Intentions, beliefs about consequences, behavioural regulation |

Additional File 3.6: Facilitators to Care at Acute/Severe Treatment of Agitation and/or Aggression Among PLWD in LTC

| **Theme** | **Code** | **Quote** | **TDF Domain(s)** |
| --- | --- | --- | --- |
| Facilitators for non-pharmacological treatments for acute agitation | Having non-pharmacological options available such as verbal de-escalation, wait and re-approach, and redirection can be critical for acute or severe agitation | At times you might want to isolate that person from the rest of the group, or from a certain person who's causing them agitation. So give them a space of their own to be in. Hopefully, maybe they can start to bring themselves down if you get rid of some of the stimulants or agitation agitators that are around in the environment. (Participant 10)  Seeing if somebody can be redirected, especially if there's somebody that is more successful with that individual. So if staff are around and somebody's particularly agitated, then that might involve having somebody who's more successful with that individual, take them out of that space. And so take them to a place where they won't hurt others or themselves. (Participant 8)  I mean, long term care, it's quite limited. I would say very worst case scenario you call paramedics or police for help. We don't physically restrain patients in long term care. So that's not something we would do. And then, I mean, just sort of verbal behavioral de-escalation is the other thing but people who are that agitated, it provides a significant challenge to long term care staff for sure. (Participant 17)  Usually, we'll try to back away from the resident - give them their space. Decrease stimulation, use a calm approach, give them time to calm down before reapproaching because when they're acutely agitated, it's difficult to argue with someone or convince them of something. So they need that time to come down from that acute agitation. (Participant 15) | Behavioural regulation,  reinforcement |
| Facilitators for pharmacological treatment for acute agitation | Using chemical restraints (i.e. medications) because  agitation symptoms are too severe (e.g. safety concerns) | When a person is in that extreme agitation [...] you've determined that this is the immediate course of action [...] to get Haldol [or] Seroquel, whatever, into that person. (Participant 10)  Well, I have a huge fear that [the resident's] going to hurt someone, right? And 98% of the time, [they] can be redirected. But that other 2%, without the medications that [they're] on, they wouldn't be able to redirect. (Participant 1)  And sometimes [...] the need is present and urgent. And so we need something to work quickly because somebody else will get hurt if we don't act sooner. And so that can be another reason. (Participant 8) | Skills Behavioural Regulation, Goals, Intentions, Beliefs about consequences |
| Facilitators for pharmacological treatment for acute agitation | Using chemical restraints (i.e. medications) because agitation symptoms do not respond to other interventions | If there's like no other option. So I can think of a resident who's kind of agitation and outbursts are almost impossible to trace to a particular need. [...] It's so difficult to know what [their] needs are [...] or if it's just something else. And so then, you know, in situations like that, the pharmacological [are] more relied on because nothing else seems to be working. (Participant 8) | Beliefs about consequences,  goals, intentions, behavioural regulation |
| Facilitators for non-pharmacological treatments for acute agitation | Having a least restraint policy | we have a least restraint policy, so physical holding of the arms and […] mechanical restraint(s) (Participant 9) | Goals, Beliefs about consequences |

Additional File 3.7: Barriers to Care at Mild/Moderate Treatment of Agitation and/or Aggression Among PLWD in LTC

| **Theme** | **Code** | **Quote** | **TDF Domain(s)** |
| --- | --- | --- | --- |
| Barriers to using medications | Interactions with disease, drugs and foods can be barriers to using medication (biological mechanisms) | I mean, there's always the disease, disease, disease-drug, drug-drug interactions, food interactions that you have to actually be aware of. And I mean sometimes, the patients or their agents have absolutely, you know, "we don't want to do this". (Participant 3)  As to which exactly we're going to choose, there are times where it's just drug-drug interactions [...] or cardiac, will [...] say no, you can't do [that] there's [...] contraindications. (Participant 3) | Knowledge, Beliefs about capabilities |
| Barriers to using medications | Severity of agitation can be a barrier to the use of some medications. | if it's severe enough, it's not as big a barrier, but it can be a barrier to management pharmacologically. (Participant 3) | Knowledge, Beliefs about capabilities |
| Barriers to using medications | Drug shortages and availability can be a barrier to the use of some medications | it's shortages, availability. What's authorized, what's not? Those are usually the the bigger barriers now for the pharmacological. And the only other one again, it's the beliefs of the residents and their agents. (Participant 3) | Environmental context and resources,  social influences |
| Barriers to using medications | Families or caregivers may not want medications used for the resident | And then sometimes family preference like families not wanting us to be using any sort of sedate medications can also be a barrier. (Participant 17) | Social/professional role and  identity |
| Barriers to using medications | Challenges in identifying side effects from the drugs | Challenges in administration. Challenges if there is not enough monitoring to see the effects of these drugs. Challenges in explaining to the caregivers what to look for in terms of side effects or other effects from the drugs. (Participant 6) | Beliefs about capabilities, environmental context and resources, social influences |
| Barriers to using medications | Challenges in monitoring medications (i.e. no monitoring of medications) | Challenges in administration. Challenges if there is not enough monitoring to see the effects of these drugs. Challenges in explaining to the caregivers what to look for in terms of side effects or other effects from the drugs. (Participant 6)  They didn't do any, but I would say there should have been. (Participant 5)  I've never seen anything, they've never told me […] I don't know if they have a 1-10 agitation level scale […] I have no clue if they do that. (Participant 2) | Beliefs about capabilities,  environmental context and resources, social influences, reinforcement, intentions |
| Barriers to using medications | Challenges in physically  administering medication (e.g.; medication administration can be traumatizing for a PLWD) | If a person is super duper agitated, they're not likely going to take a pill. (Participant 10)  People who refuse to take medications. That's probably the biggest difficulty. (Participant 7)  So that initial process to get an injection into a person, or if you have to call EMS, or call the police, or whatever it is, is very traumatizing to the individual. I mean, it does get them out of that extreme agitated state, but then [...] once they come down, you have to have [...] a period where you review that whole process with the person and go over 'How are you feeling? What was that like for you', and you have to get a briefing with the team, [...] it's in that instance it can be very traumatic for everybody involved. (Participant 10) | behavioural regulation, environmental context and resources, emotion |
| Barriers to using  non-pharmacological approaches | Comorbid neuropsychiatric diagnosis can conflict with treating agitation symptoms | So I've had a few people that way [...] it's not their dementia, it's [their] underlying paranoia. And it's been a long standing, but the dementia can sometimes just destabilize them enough that they may have compensated for 10 years or whatever without, now they're actually needing pharmacological management to help them cope. (Participant 3) | Memory Attention and Decision Processes, Emotion, Behavioural regulation |
| Barriers to using medications | Lack of education among friend and family caregivers on drug approaches for agitation and aggression | None. Got none from the nursing home at all, no. (Participant 5) | Social/professional role and  identity, knowledge |
| Barriers to using  non-pharmacological approaches | Lack of non-pharmacological interventions available for  agitation or aggression | We've discussed the options I've put forward, but they haven't put forward anything. (Participant 2)  I'm feeling like the options that they're using are the ones that I've brought to them. Like, I'm not feeling like they really have a system for that or [...] I would expect to see a checklist of different things you could try, right? But I'm feeling like, I'm bringing them the options rather than them saying, okay, these are some of the things we can do. (Participant 2) | Environmental context and  resources, social influences, knowledge, skills |
| Barriers to using  non-pharmacological approaches | Lack of training specifically for non-pharmacological treatment approaches among LTC staff | I wish more people would try the non-medicinal interventions and also that there is more resources towards training the caregivers in understanding non-medicinal interventions and how they can be beneficial. I think they are quite beneficial in most cases, for most people. (Participant 6) | Skills, knowledge |
| Barriers to using  non-pharmacological approaches | Difficulty coordinating timing for intervention among a group of residents (E.g. reluctance to participate in non-pharmacological activities) | So we can provide a seated exercise group and we can provide a cognitive group, but [...] when is the right time? Because maybe 'Joe' and 'Becky' are ready for it, but your other residents aren't. They're distracted and off you go, you still don't have a group. (Participant 9)  [The resident] may refuse to come out of [their] room, I think is what happens. I know, they do have some music therapy. [...] I've met that therapist [...] and she's said she'd never had [the resident come] to a program [...] (Participant 4) | Environmental context and resources, social influences, memory attention and decision processes |
| Barriers to using medications | Poor response or worsening of behaviour when medications were used | They had [the resident] medicated in [LTC centre], and it just made it so much worse. I had to basically demand that they at least cut it back. And when they cut it back, they found [the resident] improved. (Participant 5)  Unfortunately, [the resident] was one of those people that did not handle [medications] well. (Participant 1) | Beliefs about consequences, behavioural regulation |
| Barriers to using  non-pharmacological approaches | Reliance on medications | In the first facility, they've tended to overmedicate, just to make [the resident] sleep [...] a lot of Trazodone [...] as needed. [...] They switched [their] anti-psychotic [...] to carbamazepine. Again, a type of antipsychotic. [...] And then they added another antipsychotic, Seroquel. (Participant 1) | Beliefs about consequences, behavioural regulation |
| Barriers to using  non-pharmacological approaches | Advancement in dementia results in frequent changes in non-pharmacological treatment plan needed | The non-pharmacological stuff takes time to figure out. And [...] as dementia advances, it changes over time. And so somebody [...] needing short term, that might be three to six months, of a particular type of thing, [...] may be feasible, or may be not. (Participant 8) | Memory Attention and Decision Processes, Social influences, environmental context and resources |
| Barriers to using  non-pharmacological approaches | Risk of using non-pharmacological approach (e.g. behaviour does not improve) | The risk could be that if it doesn't work, you might still be faced with the behavior. (Participant 7) | Beliefs about consequences |
| Barriers to using medications | Adverse side effects of medications | Using drugs can make [the resident] zombie-like, that's the big thing, and not everybody responds to a specific drug the way it's intended to work (Participant 1)  Yes, because the pharmacological ones have side effects, right? And so [...] think of how [that] pharmacological intervention affect[s] them physically [...] always trying non pharmacological is going to be the best option. (Participant 9) | Beliefs about consequences, behavioural regulation |
| Barriers to using medications | Loss of personal traits or skills after administering medication for agitation | Once they have their drug plan in place, they're very much not as aware of their environment as they were before they started taking the drugs. I have people that before they were on the drugs, they were walking around, they were interacting, you hardly knew that they even had dementia. And then once they're on the drugs, they're in a wheelchair, they can't really communicate very well. And[...] they don't always sense even what's around them in the environment. So just like such a drastic change, and not who they are. (Participant 7) | Emotion, memory attention and decision processes |
| Barriers to using  non-pharmacological approaches | Use of medication because it  is convenient | I think we are all used to quick fixes and think that drugs, the quick fixes to any issues you might be having. (Participant 6)  The default is drugs, [...] agitation and aggression can be managed with other things other than drugs, but because drugs are the easiest. Given the staffing shortage, it seems to be the default. (Participant 6)  I think sometimes the pharmacological is easier way to go. Considering also like how much funding we get for staff, and how much time they have to actually do the non-pharmacological interventions. I think that's just kind of what works for our healthcare system. (Participant 7) | behavioural regulation, environmental context and resources, social influences |
| Barriers to using medications | Not all types of agitation are  responsive to medications | There's a lot of times where, certainly for agitation, pharmacological won't necessarily do a whole lot. So there's just certain things you just can't treat with medication- calling out or spitting, or things you just can't... there's no pill that will fix that. (Participant 10) | Environmental context and  resources, behavioural regulation, emotion |
| Barriers to using  non-pharmacological approaches | Easy to access prescriptions for agitation medications | No, no there never [were barriers to accessing medication]. Maybe there should have been more, particularly the antipsychotics? [...] Maybe like, don't give them a prescription for 30 of them. (Participant 1)  Medications are all covered, they come to the facility. Easy. (Participant 4) | Environmental context and  resources, social influences |
| Barriers to using  non-pharmacological approaches | Needing to use trial and error to  choose non-pharmacological approach | I think we just kind of trial the different ones and see what works. So it's kind of trial and error. Also asking family for some input. (Participant 7)  And so, a certain amount of it is then aspects of like, trial and error, exploring what might work and again (Participant 8) | Skills, knowledge, beliefs  about consequences, goals, intentions |
| Barriers to using  non-pharmacological approaches | Lack of sensory experience non-pharmacological approaches | Thinking about the ways that we can incorporate scent into a place and just how we naturally experience a variety of scents throughout our day, or, you know, even within a safe space [...]. Oftentimes, in a lot of units, you might get the shower scents, or you might get the bowel movements scents. But like there isn't always as much variation. [...] So looking at the different ways - or even nature scents, how do we get people that exposure? (Participant 8)  Getting to use those sensory experiences along with body movement, I think [...] have neat potential for at least some residents at times. (Participant 8) | Intentions, goals,  environmental context and resources |
| Barriers to providing general  treatment for agitation and/or aggression | Inconsistent monitoring of  interventions | It's not consistent. Definitely something to work on. (Participant 12) | Reinforcement, beliefs about  consequences |
| Barriers to using  non-pharmacological approaches | Staff are afraid to use  non-pharmacological interventions | Sometimes that depends on the staff's perception of the situation,  they may not be comfortable approaching the resident, they may feel afraid. (Participant 15) | Skills, social/professional role and  identity, beliefs about capabilities |
| Barriers to using  non-pharmacological approaches | Non-pharmacological interventions are only administered by nursing staff, not physicians, thus barriers to use are not known by physicians | Well, [family physicians] don't administer that. That's nursing and  nursing is an independent profession. So they make nursing plans, because they're facing the same issue that we are. So we do our part, they do their part, we don't tell them what to do. (Participant 16) | Social/professional role and  identity, knowledge |
| Barriers to using  non-pharmacological approaches | Staff pressures on physicians to move to medication sooner | But I would say sometimes families' preference will influence one  way or the other. Sometimes pressure from staff - there certainly are times when my preference would be to not jump straight to medications, but staff are telling [me] that they just can't manage somebody [...] because of whatever staffing issues. So there's times when that will influence my decision to go to medication sooner than later. (Participant 17) | Social influences, environmental  context and resources, social/professional role and identity |

Additional File 3.8: Facilitators to Care at Mild/Moderate Treatment of Agitation and/or Aggression Among PLWD in LTC

| **Theme** | **Code** | **Quote** | **TDF Domain(s)** |
| --- | --- | --- | --- |
| Facilitators to using medications | Easy to access prescriptions for agitation medications | No, no there never [were any barriers to accessing medications]. There never was any. Maybe there should have been more, particularly the antipsychotics? [...] Maybe like, don't give them a prescription for 30 of them. (Participant 1)  Medications are all covered, they come to the facility. Easy. (Participant 4) | Environmental context and  resources, social influences |
| Facilitators to using  non-pharmacological interventions | Routine monitoring of  non-pharmacological approaches | So we have a book that's set up in [the resident's] room. And so my recreation therapist writes her case notes after every visit. And then the family is able to review them. So she said, she'll write about what they did, how he reacted. Any issues, any signs of agitation, and also she'll just write in a plan for their next visit. (Participant 4)  I think the hidden charts where they monitored what was happening after, you know, the interventions, and then these would be discussed at the next family meeting. (Participant 4) | Behavioural regulation,  reinforcement, intentions, goals |
| Facilitators to using medications | Routine monitoring of  medications | I think it was through the daily monitoring. It's how they get it, and they reduced it slowly. [...] Because their principle is not to over medicate. (Participant 1)  If it's pharmacological, you can use your BSMTs to determinate if there's a behavior shift or change. Sometimes if it's pain and you add medication for pain, then you can determine behavior or see decreased agitation (Participant 10) | Behavioural regulation,  reinforcement, intentions, goals |
| Facilitators to using medications | Seeing the patient improve with medication (E.g. making patients more content) | I was happy with the medication. That was my number one concern was that, you know, knowing that [the resident] didn't know where [they were] most of the time, didn't know what was happening, and this would have increased all this agitation [...], and I just really wanted [them] to feel comfortable. (Participant 4)  [The resident] spent three months at [hospital] waiting for placement. And there was no interventions besides restrain, [...] and when [they] moved to [LTC centre], then they started using the medications at the other modalities. There was a difference. [They are] happier [...] and not as aggressive. (Participant 6) | Beliefs about consequences,  optimism, goals, intentions |
| Facilitators to using  non-pharmacological interventions | Positive outcomes from non-pharmacological treatments for agitation | Like if he is agitated by a particular resident being noisy or what not, he'll walk to his room. So [...] it has helped that, he hasn't had a physical altercation in two months. (Participant 1)  They've suggested music therapy, which does help. And they do. Like even the physical therapy and cognition therapy that they do helps a lot because it keeps the mind working and not going to dangerous places (Participant 5) | beliefs about consequences, optimism, behavioural regulation |
| Facilitators to using  non-pharmacological interventions | Intentional use of non-pharmacological  treatment strategies | They use different activities - recreational activities. If [the resident] starts to get agitated during the summertime, they would take [them] outside to they have an outdoor enclosed area that would calm [them] down. [...] So they would try to redirect [them] with activities. (Participant 1)  We always try our best to redirect them [...] to intervene, to do something for them when they're agitated. (Participant 13) | Environmental context and resources |
| Facilitators to providing general treatment for agitation and/or aggression | Gentle Persuasion Approach taught among staff | So again, our GPA training that we have in our building. Everybody has that knowledge and skill set in [their] back pocket. (Participant 10)  We practice the gentle persuasive method here. And so, it's approaching the client with everything we've talked about, trying to reduce the agitation in the beginning, and the aggression, so that it doesn't escalate. Because we do have training when it's escalating on how to do nonviolent crisis intervention (Participant 9) | Skills, Knowledge |
| Facilitators to using  non-pharmacological interventions | Having familiar and developing trust with healthcare providers each time to administer non-pharmacological support for residents | Having staff [become] familiar with residents because then they get to recognize when agitation's coming and also see some of the patterns for the behaviors - what does this person respond well to, what did they not respond well to? So that over time the families may be providing initial information but staff have come to really know an individual, and predict some more of those things. (Participant 8)  So being able to develop trust is, to me, is one of the biggest things in any sort of any profession, no matter what intervention you're trying to provide, whether it be rec therapy or OT, relieving comfort and discomfort and pain, or nursing, providing pharmacological or whatever. If you can't develop a trusting relationship with that person, either over time or in the moment, then your intervention isn't likely to work. (Participant 10) | social/professional role and identity, reinforcement |
| Facilitators to using  non-pharmacological interventions | Choosing non-pharmacological approaches as first line | So there certainly is a place for the non-pharmacological interventions. And they should be tried first. It's the least invasive for the person (Participant 10)  Absolutely non-pharmacological first step, and the first way to go and I'm reasonably patient where it's trying that non pharmacological management. (Participant 3)  If you can avoid the pharmacological treatments, you're way better off. (Participant 5) | Optimism, beliefs about consequences, beliefs about capabilities |
| Facilitators to using  non-pharmacological interventions | Specifically assessing basic needs as first line non-pharmacological treatment | First and foremost, figuring out if there's actually a need, like if they're hungry, thirsty, those basic needs need to go the bathroom needs to be changed, and pain. [...] There's five assessments we look at [...] (Participant 9)  It's an, in the moment, we have to kind of decide for each person, what is this person experiencing right now? And what are the likely factors? Have they had a recent fall? Have they had a recent change in medication? Have they had a recent loss or is there grief involved? So I'm just trying to identify every aspect of their lives, to determine what may be the likely factors and go from there and plan for that. (Participant 10) | Goals, intentions, beliefs about  consequences, skills, knowledge |
| Facilitators to using  non-pharmacological interventions | Ensuring staff have the competence and training to administer non-pharmacological treatment approaches | We have seen them in action, providing some of these interventions to other patients. And I feel that they seemed comfortable in what they were doing. So I think they have the education to be able to provide what they're providing. (Participant 6)  [I]mmediately when [resident] entered [LTC centre], they told me about it. They sent a questionnaire about what type of music she liked. What the things were to play in that. They have separate players, earphones and everything (Participant 5) | Skills, Knowledge, Beliefs about capabilities |
| Facilitators to using  non-pharmacological interventions | Treatment for agitation depends on the confidence and education of staff to administer non-pharmacological interventions | The person has to have the confidence and the ability and the skill and the knowledge to see where this person is at in their agitation. And how can I first look at reducing without that pharmacological, so what skills and strengths do I have to get through this in the moment, right now? What's the fastest path to success? [...] Are they in that extreme, dangerous environment where we're beyond nonpharmacological? And if we're in that non pharmacological range, how skilled is the person to be able to [handle] that? (Participant 10) | Skills, Beliefs about capabilities |
| Facilitators to using medications | Use of medication because it helps address agitated behaviours related to dementia | It's always with any medications, pharmacological interventions address certain areas of behavior and cognition related to dementia. (Participant 9) | Skills, beliefs about consequences, behavioural regulation |
| Facilitators to providing general treatment for agitation | No regular guidelines to use restraints for agitated patients | They don't use any restraints whatsoever. They sometimes just put [the resident] in the quiet room with somebody watching, make sure she doesn't harm herself and medication. (Participant 6) | Intentions, goals,  environmental context and resources |
| Facilitators to using medications | Although doctors prescribe, the whole interdisciplinary team reports on the effectiveness of treatments | How they're actually prescribed is, it becomes the doctor's orders, ultimately, but the doctor does rely on feedback from the nursing staff as well on what's been effective or not. (Participant 9) | social/professional role and identity |
| Facilitators to using medications | IM administration route eases ability  to administer medication | And I mean, if it is IM injection or so, then, sure they have their certain [...] approaches to do that. And I think this is then more successful (Participant 12)  Acute and severe I would say, usually medication and often in that situation, I'd be looking at an injectable medication if they won't take an oral so, you know, an IM medication of some sort if it's that severe. (Participant 17) | Skills, Goals, Intentions,  behavioural regulation |
| Facilitators to providing general treatment for agitation and/or aggression | Using documentation to  monitor interventions | I would say mostly the documentation to really demonstrate, okay, so this intervention was used, and it was effective, and then also with communication, doing staff meetings, and so really to say, 'Okay, now, this intervention worked very well that day, and I just come in whenever with this resident, it works perfect, why don't you try it'. (Participant 12)  Very closely, and we have the staffing notes, we have the physician notes, I write a note each week with the factors that we're focused on. Again, we have things like the depression score, the chess score, the rugs, the RAI inputs - it all can come together, but somebody has to look at it and make sense of it. And that person is typically a nursing administrator. (Participant 16) | Behavioral regulation,  reinforcement |
| Facilitators to using medications | Some residents do respond well  to medications for agitation and/or aggression | Some behaviours specifically do respond to medication. Or sometimes someone  has been on a medication for a long time. And it's worked for them, so it's preferred. When they come of of it, their dementia, their functioning becomes worse. So sometimes the medication really does help them. (Participant 15) | Behavioural regulation, Intentions, Goals, Optimism |
| Facilitators to providing general  treatment for agitation and/or aggression | Best treatment approach is  dependent on the person (drug vs. non-drug) | Depends on the resident, because some residents [...] the non-drug approach works best, works faster. But then if they're really [...] aggressive and agitated, then the nurses might need to administer that drug interventions. (Participant 13)  Yeah, sometimes that depends on the person. Sometimes [non-pharmacological interventions] can be even more effective. It just sometimes takes more time, and more resources that we may have at the time, but it depends on the situation [and] on the person. Some things really work for them. You may have to do it several times throughout the day, so that's kind of a constant thing. (Participant 15)  I don't have an answer to that question because I don't think you can ask that question fairly. It's all part of a compendium of options, and you need to use the appropriate one at the appropriate time for the appropriate person. (Participant 16)  But each one has its has its place, and it really depends on the situation. (Participant 10) | Skills, Knowledge, Goals,  Intentions |
| Facilitators to using  non-pharmacological interventions | Resources are available that support the use of non-pharmacological interventions (e.g. geriatric mental health) | Our ABC team and geriatric mental health - they do give us suggestions on non-pharmacological [approaches]. (Participant 15)  I'd say the geriatric mental health or the clinical nurse specialists are a great resource, but they're not as available as we would want them to be. And then, you know, when facilities have the ability to augment staffing to help with some of these higher agitation residents, that can be very effective (Participant 17) | Environmental context and  resources, social influences |
| Facilitators to providing general treatment for agitation | Ensuring plans are in place to reassess residents to potentially deprescribe  medication | [W]e like to reassess every three months. Look at whether or not deprescribing is possible (Participant 16)  And the other thing is, sometimes we find people come in, they've got all these drugs from the community, but they haven't got the community problems anymore. So we try and tidy things up, keep it simple. Use the tools you've got, which maybe they didn't have in the community. (Participant 16) | Reinforcement, Behavioural  Regulation, Beliefs about consequences |
